# SpinachXAI-Rec: a multi-stage explainable AI framework for spinach freshness classification and consumer recommendation

**DOI:** 10.1038/s41598-025-19804-y

**Published:** 2025-10-14

**Authors:** Akella S. Narasimha Raju, G. Sujatha, Ranjit Kumar Gatla, Shilpa Ankalaki

**Affiliations:** 1 Department of Computing Technologies, School of Computing, College of Engineering & Technology, SRM Institute of Science and Technology, Kattankulathur, Chennai, Tamil Nadu 603203 India; 2 Department of Networking and Communications, School of Computing, College of Engineering & Technology, SRM Institute of Science and Technology, Kattankulathur, Chennai, Tamil Nadu 603203 India; 3 Department of Computer Science and Engineering (Data Science), Institute of Aeronautical Engineering, Hyderabad, Telangana 500043 India; 4 Manipal Institute of Technology Bengaluru, Manipal Academy of Higher Education, Manipal, India

**Keywords:** Spinach freshness classification, DenseNet121, Vision transformer (ViT), Explainable AI (XAI), GradCAM++, LIME, Deep feature embeddings, Rule-based recommender system, Computational biology and bioinformatics, Health care, Mathematics and computing

## Abstract

Leafy vegetables such as spinach are among the most important components in a nutritious diet but are highly perishable and susceptible to premature spoilage. Traditional practices in determining freshness have been qualitative and time-consuming and have consistently led to defective consumption decisions with unintended consequences on human health. To this issue, we introduce SpinachXAI-Rec, a multistage framework that is enabled by AI and is capable of automating the classification of spinach freshness and providing consumer recommendations. This framework is based on understandable deep learning. To guarantee class balance and feature diversity, a dataset consisting of 4005 original images of three spinach varieties (Malabar, Red, and Water) was expanded to 12,000 images (2000 per class across six categories: fresh and non-fresh). We trained three CNN architectures, DenseNet121, ResNet50, and EfficientNetB0, on the Stage 1 augmented dataset. In performance, we saw DenseNet121 significantly outperform with 96% classification accuracy compared to ResNet50 (53%) and EfficientNetB0 (17%). Stage 2 improved representation of features by incorporating DenseNet121 embeddings and ViT-B/16 and Swin Transformer attention mechanisms. DenseNet121 + ViT-B/16 obtained an F1-score of 0.95, which was further optimised to 0.97 in Stage 3 using a multiclass SVM classifier. GradCAM++ and LIME were employed to incorporate interpretability during Stage 4. LIME provided transparent explanations of the significance of class-specific features, while GradCAM++ effectively highlighted disease-affected or spoilt regions. The most effective model (DenseNet121 + ViT + SVM) also obtained a Dice coefficient of 0.89 and an IoU of 0.82, which confirms the precision of localisation and segmentation. Finally, Stage 5 introduces a clinical recommender system that is based on rules and relates prediction confidence to real-world categories: Eatable, Eatable with Caution, or Not Eatable. This AI-driven recommendation assists food purveyors and consumers in making health-conscious, well-informed decisions. SpinachXAI-Rec is a significant advancement in the development of safer food systems, as it provides interpretable AI for the purpose of freshness validation and actionable consumption recommendations, thereby empowering both consumers and industry stakeholders.

## Introduction

Internationally, people recognise green leafy vegetables (GLVs) as essential components of a healthy diet, offering a wide range of physiological benefits. GLVs play a key role in the prevention of lifestyle disorders like osteoporosis, diabetes, cardiovascular diseases, and anaemia^1^. These compounds include a variety of bioactive substances such as flavonoids, carotenoids, and micronutrients like iron, calcium, folate, magnesium, and fibre^2,3^. Spinach is a “superfood” with the highest therapeutic effects and nutrient content among the GLVs^4^. China was responsible for the production of more than 92% of the world’s spinach in 2022 and produced well over 30 million tonnes, says the Food and Agriculture Organisation (FAO). Second and third places, respectively, were held by the United States and Japan. The global significance of this crop product is the growing interest in spinach among urban communities in particular that are health-conscious in being provided in smoothies, salads, and organic versions^5^. However, the perishability owing to the water content and delicately foliated nature of spinach results in widespread losses and qualitative losses en route and in storage. This loss presents a great challenge to ensuring the end-consumer with safety and freshness. The quality inspection regimes at present are still almost completely manual, unscalable, and qualitative and therefore pose a great need for automated, quantitative, and interpretable AI-powered freshness determination tools.

Spinach is of significant cultural, dietary, and agricultural importance in the Indian context. According to the Ministry of Agriculture & Farmers’ Welfare (2021–22), India is one of the top five spinach producers, with an area exceeding 150,000 hectares and a contribution of approximately 1.7 million metric tonnes^6^. Traditional Indian cuisine is profoundly influenced by spinach and its indigenous varieties, including Malabar spinach (Basella alba), red spinach (Amaranthus dubius), and water spinach (Ipomoea aquatica)^7^. They are essential components of daily diets, particularly among the vegetarian population, which comprises more than 30% of India’s population^8^. Their effects are maximised in Southern India, where the production and consumption fronts are led by the states of Tamil Nadu, Andhra Pradesh, Karnataka, and Kerala. These states’ favourable agro-climatic conditions enable year-round plantings. Apart from this, physically fresh and safe-for-consumption spinach demand has increased with urban market expansion, organic farms, and home delivery of vegetables^9^. However, the evaluation of freshness at retail stores remains error-prone and manual, which negatively impacts supply chain efficiency and end-consumer confidence. Therefore, to have any advantage for growers, retailers, and health-conscious consumers in all of India, an intelligent, comprehensible, and robust solution has to be designed to evaluate and suggest the freshness of spinach^10^.

In both urban and rural channels of distribution, categorization of spinach as eatable or non-eatable remains a troublesome and unresolved problem in spite of its widespread use and nutrient importance. Deteriorations caused by moisture loss, microbial spoilage, physical damage, and discolouration of the leaves collectively lead to a rapid decline in the visible external appeal of spinach^11,12^. However, these deteriorations are often qualitative and subtle, making traditional manual checks highly inconsistent and prone to errors. Given the potential for conflict and losses in products and money, something permissible to one vendor is rejected by one quality checker or consumer due to the risk of conflict and losses in money and products. Moreover, in today’s supply chain operations, no standardised method or objective tool exists to quantify and decide upon the freshness limit at which spinach shifts from being safe to being unsafe to eat^13,14^. Determinations at retail stores and open markets and doorstep delivery points remain purely based upon human perception and compound these inconsistencies while raising questions regarding food safety and trust and assurance regarding quality.

It is important to create intelligent, automated, and understandable classification processes that can categorise spinach leaves as eatable or non-eatable reliably to address this long-standing problem^15^. Powerful extraction of features from high-resolution spinach leaves’ images is made possible through capitalising on the power of deep learning and artificial intelligence (AI), foremost among them being convolutional neural networks (CNNs) and vision transformers (ViTs)^16,17^. AI models are empowered to learn to recognise small visible cues regarding quality in the leaves by being trained upon large sets of labelled data demonstrating things like colour transformations and wilts and number of holes and textural damage^18–20^. However, grading is not merely classification; grading must offer interpretability and trust and clinical rationale as well, notably when the result has repercussions for decision-making in terms of consuming the foods. As a result, embedding confidence-based suggestion schemes with explainability AI tools like GradCAM++ and LIME aid in the development of a system like an expert’s judgement in clinical terms^21^. The goal is to enable transparent, scalable, and intelligent freshness determination at the consumer level by regulating quality detection and providing suggestions about the usability of spinach leaves for consumption.

### Motivation and problem statement

The perishable nature of spinach, a nutrient-rich and extensively consumed verdant vegetable, presents a significant challenge in post-harvest quality assessment. The subjective, inconsistent, and inefficient manual classification of spinach freshness into eatable and non-eatable categories is a problem that persists throughout retail and supply chains. Manual graders usually overlook or misread fine visible deteriorations like discolouring, withering, and damaged leaves. This non-standardisation has direct economic and food safety ramifications. Therefore, we very much need an automated, interpretable and intelligent system to correctly classify and estimate spinach quality based on visually apparent signals. A possible remedy to this issue is to use AI, particularly deep learning and interpretable models, to predict freshness in a transparent, scalable, and clinically applicable manner.

Due to their nutrient-dense and health-promoting properties, green leafy vegetables, and in particular spinach, are constituents of any balanced diet. Nevertheless, their rapid spoilage upon harvest and lack of explicit means to classify them make it difficult to preserve these products in a fresh and safe condition for consumption.

SpinachNet-XAI Framework Objectives

1. We have expanded the original set of 4005 images to 12,000 images to create an augmented image dataset of spinach leaves. This dataset encompasses six classes across three spinach types (Malabar, red, and water spinach) and fresh and non-fresh conditions.
2. To classify spinach freshness and determine the optimal architecture based on accuracy, precision, recall, and F1-score, various deep learning models (DenseNet121, ResNet50, and EfficientNetB0) are implemented and evaluated.
3. To improve performance, this research suggests a hybrid classification pipeline that incorporates deep features of DenseNet121 and Vision Transformer (ViT) with an ensemble SVM classifier to further improve accuracy and provide improved generalisation.
4. To improve the interpretability and trustworthiness of the classification outcomes, it is necessary to incorporate understandable AI techniques, specifically GradCAM++ and LIME, to visualise the regions that influence model decisions.
5. To have consistent and dependable explainability in freshness evaluation, segmentation metrics IoU and Dice coefficient are computed, resulting in an IoU of 0.89 and a Dice coefficient of 0.93.
6. This research proposes developing a clinical recommender system based on rule-based concepts to categorise spinach as eatable or non-eatable through prediction confidence, visual explanations, and threshold logic to help consumers and vendors make better decisions.

## Literature survey

Table 1 delineates recent (2022–2025) peer-reviewed publications on deep learning and computer vision to quantify vegetable and leafy green quality and freshness. Some selected publications include fully reviewed articles instead of preprints and include explicit measurement of accuracy. They demonstrate excellent performance with bespoke CNNs and hyperspectral-ML integration but do not fully encapsulate the concurrent necessities for spinach-orientated training, explainability (XAI) outputs, and end-consumer eatability recommendations. This points to the innovativeness and contribution of SpinachNet-XAI. Table 1 presents the detailed literature survey.

**Table 1:** Literature survey.

Author(s)	Year	Publication	Methodology	Accuracy	Advantages	Limitations	Research Gap
Sankar Sennan et al^22^	2022	*Computers, Materials & Continua*	Custom CNN on four leaf types	97.50%	High accuracy; compared multiple baselines	Small (~ 400 images); non-spinach types	No XAI; limited generalization
Yıldırım and Yalçın^23^	2024	*J. Food Nutr. Res*	ResNet-101-based CNN for spinach freshness	≥ 89.4%	Good baseline for food quality tasks	Limited accuracy; no interpretability	Absence of XAI; no hybrid ensemble
He et al^24^	2024	*Infrared Physics & Technology*	Hyperspectral + DL classifiers (spinach + cabbage)	> 80%	Non-destructive biochemical analysis	Expensive instrumentation; lower accuracy	No image-CNN/XAI; equipment-heavy
Kumar et al.^25^	2024	*Current Research in Food Science*	CNN–BiLSTM hybrid for generic vegetable freshness	97.76%	Models spatial & temporal features	Computationally heavy; generic to veggies	Not spinach-specific; lacks visual explanations
Tapia-Mendez et al^26^	2023	*Applied Sciences*	MobileNetV2 ensemble for fruit & vegetable ripeness	97.86%	High accuracy across ripeness stages	Broad domain; no spinach focus; no XAI	No spinach dataset; no explainability
Yuan & Chen^27^	2024	*Current Research in Food Science*	Deep feature fusion (GoogLeNet, DenseNet-201, ResNeXt-101) + PCA + SVM	96.98%	No CNN retraining; efficient feature-based detection	Not spinach-specific; image quality threshold unclear	No explainable visualization
Koyama et al.^28^	2021	*PLOS ONE*	Color & local feature + SVM/ANN for spinach freshness	84% (2-class)	Non-destructive; validated against sensory panel	Lower accuracy; no deep learning	No image-based DL; no XAI; training on small smartphone dataset
Elumalai and Meganathan^29^	2024	*J. Robotics & Control*	Hybrid of Orange-embedded pre-trained models + ML classifiers on spinach leaves	~ 99%	High accuracy; variety classification	Limited information on freshness; tool-specific	No interpretability; no confidence-based recommendation logic

### Research gap

Although various works in the past have investigated the use of deep learning and computer vision for freshness estimation in fruits and vegetables, most have considered generalised or cross-category data without fine-tuning the classifier for spinach. CNN ensembles, BiLSTM hybrids, and hyperspectral-based classifiers are advanced models that have shown accuracy rates over 95%. However, they often face major issues, such as not providing clear decision support, lacking easy-to-use visual checks for consumers, and not having explainable AI (XAI). The cost and hardware dependencies of hyperspectral methods render them impractical for field or retail use, despite their biochemical accuracy. Additionally, there is a scarcity of research that integrates confidence-based evaluation or recommendation logic, which is crucial for real-world applications such as retail quality assurance or mobile-based spinach eatability assessment. Most importantly, existing studies rarely focus on combining a rule-based clinical recommender, methods to explain decisions (like GradCAM++ and LIME), and deep classification all in one complete system. This gap creates a distinct and substantial void for a deep learning system that is spinach-centric, interpretable, and actionable—exactly the objective of SpinachNet-XAI.

SpinachXAI-Rec explicitly vis-à-vis novel leafy green classification and food quality AI-based approaches. We have also provided an additional paragraph briefly summing up the high degree of precision of the works.Authors Sankar Sennan et al. (2022), Yıldırım & Yalçın (2024), and Tapia Mendez et al. (2023). But none of those embodied essential aspects, like spinach specialisation, two-layer interpretability (GradCAM++ and LIME), or a clinically actionable rule-based recommendation system. SpinachXAI-Rec, however, achieves high performance (97.2% accuracy) and transparent, confidence-based decision semantics for real-world consumer and vendor deployments through integrating DenseNet121 and ViT-B/16 embeddings with an SVM classifier. The paper clarifies our approach’s novelty and positions it as an integrated, interpretable, and spinach-centric upgrade to state-of-the-art approaches.

### Technologies utilized in SpinachNet-XAI framework

The SpinachNet-XAI system takes advantage of a robust set of state-of-the-art technologies from deep learning, explainable AI, image processing, and clinical decision support areas. The system incorporates the following key technologies:

- Convolutional Neural Networks (CNNs
  Baseline image classification applications employ CNN models like DenseNet121, ResNet50, and EfficientNetB0. DenseNet121, with its dense connectivity and feature reuse, demonstrated the highest performance among these models, achieving 96% accuracy in classification.
- Vision Transformer (ViT):
  ViT is applied to learn long-range spatial dependencies and enhance feature representation. It is applied with features in DenseNet121 to build a hybrid deep model for features, and with the help of SVM achieves 97% accuracy in freshness classification of spinach.
- Support Vector Machine (SVM):
  The last classifier in the hybrid model pipeline is a multiclass support vector machine (SVM). This classifier accepts fused deep features (DenseNet121 + ViT) and fine-tunes decision boundaries for high-dimensional embedding features.
- Explainable AI (XAI) Methods:
  GradCAM++: Used to produce attention maps visually highlighting salient areas influencing the CNN predictions. Enabling visual confidence and clinical judgement. LIME (Local Interpretable Model-agnostic Explanations): Utilised to construct feature-importance overlays with instance-wise interpretation. These techniques provide explainability to model decisions with segmentation-level interpretability and IoU and Dice coefficient scores of 0.89 and 0.93, respectively.
- Dimension Reduction and Visualisation:
  t-SNE and UMAP are used to map high-dimensional deep feature embeddings to 2D space in order to visualise class separability and learning dynamics.
  Animated t-SNE/UMAP over training epochs helps visualise the convergence of features among classes.
- Image Augmentation & Preprocessing:
  This initial dataset with 4005 pictures is then expanded to 12,000 pictures with transformations like rotation, flip, zooming, variation in amount of light, and crop with the aim to improve generalisation and reduce overfitting.
- Clinical Recommender System:
  A rules-based decision engine takes model confidence scores, XAI visualisation signals, and threshold logic and turns them into eat-or-don’t-eat recommendations. This bridges AI output to in-the-world decision-making among vendors and buyers.
- Development Environment & Libraries
  We trained the model using TensorFlow/Keras and visualised it using Python. These experiments were conducted using Google Colab Pro+ with GPU acceleration (e.g., Tesla T4 or A100) to facilitate efficient training and inference.

### Critical perspectives: regulatory, trust, and deployment challenges

A critical review of recent food-quality vision systems shows that most works prioritise accuracy while giving limited treatment to the non-technical constraints that determine real-world viability—namely regulatory compliance, trust, and deployment robustness. Specifically, prior studies rarely map their methods to food-safety frameworks (e.g., HACCP/Codex/ISO 22000 and country regulators such as FSSAI/USDA/EU), omit calibration and uncertainty reporting, and provide no auditable trail linking model outputs to end-user actions—weakening accountability and consumer trust. They also underaddress domain shift (lighting, devices, backgrounds, handling conditions), do not specify human-in-the-loop overrides for borderline cases, and lack post-deployment monitoring for drift, latency, or failure modes. In contrast, our framework explicitly integrates conservative, risk-aware decision thresholds tied to a rule-based policy; dual-layer explainability (GradCAM+++ LIME) to support auditability; confidence calibration and error analysis to mitigate false reassurance vs waste; and a deployment pathway that includes site-specific validation, device constraints, and operator escalation for ambiguous predictions. This positions the approach as not only performant in controlled experiments but also aligned with regulatory expectations, transparent to stakeholders, and resilient to real-world operational variability.

## Methodology

**Fig. 1:**
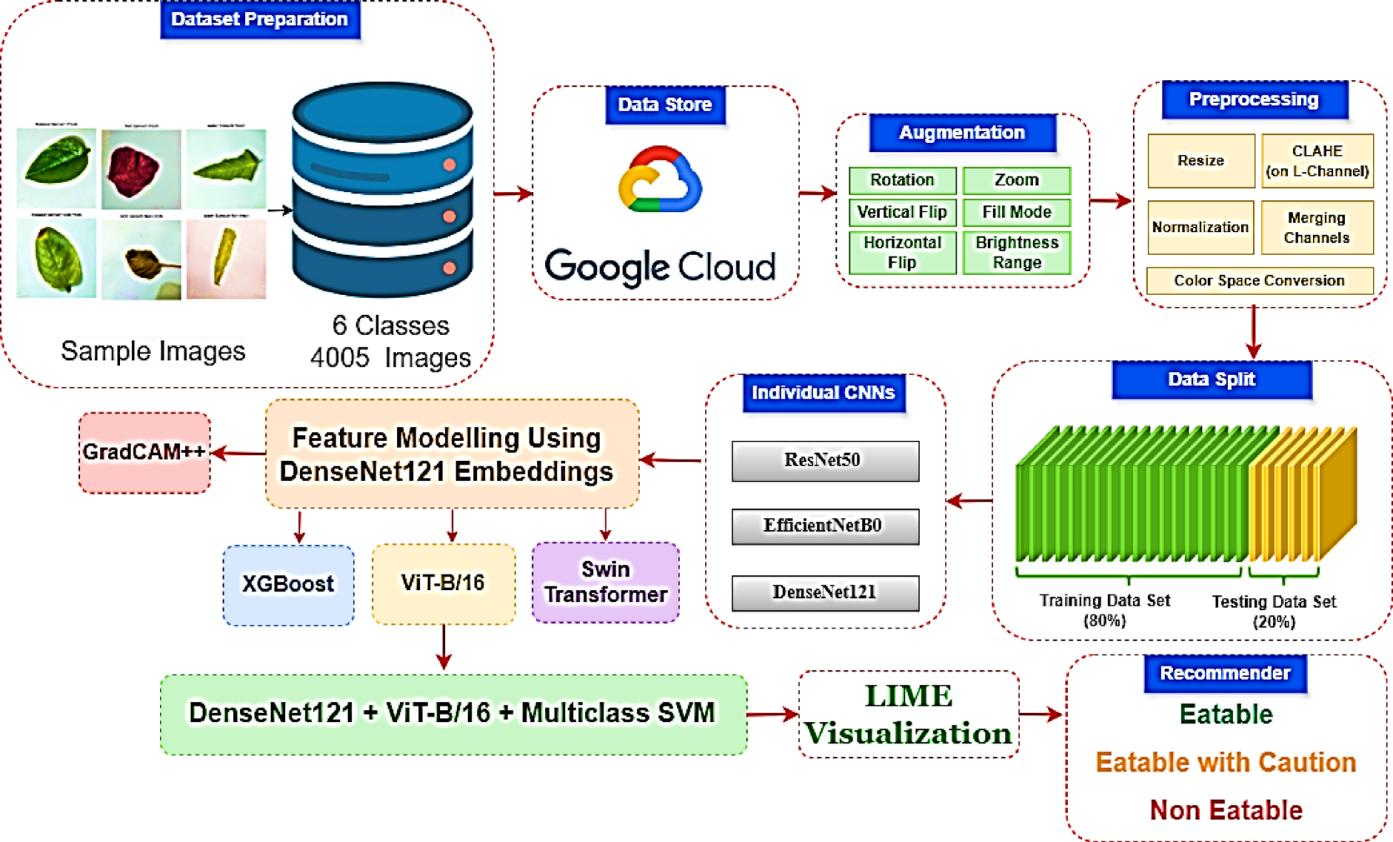
Architectural pipeline of the SpinachXAI-Rec framework.

The SpinachXAI-Rec framework is an end-to-end, six-step methodology aimed at automating the evaluation of the freshness of spinach and giving the end-user interpreted recommendations^30^. The pipeline begins with Stage 1, Data Augmentation, as depicted in Fig. 1. Here, the raw images of the spinach are augmented by the transformation to guarantee the balance of the class and generalise the model successfully. Stage 2: Baseline Classification entails training several convolutional neural networks (CNNs) to assess the network’s capacity to classify the non-fresh and fresh spinach classes. For extraction of global and local visual patterns, Stage 3: Hybrid Feature Extraction combines features by attention from vision transformers and spatial features from the best-performing CNN^31^. The features from augmentation go to Stage 4: Multiclass Classification, where the decision-making model, i.e., Support Vector Machine, is utilised to maximise the separability of the classes between the different categories of spinach and stages of ripeness. Stage 5: Explainability integrates explainable AI tools such as GradCAM++ and LIME to qualitatively validate model predictions and identify the decision-imperative regions in the images of the spinach qualitatively^32^. Lastly, Stage 6: Clinical Recommender System interprets the model’s output to the end-user advisability by mapping the model’s output to the categories in the real world, for example, the categories “Eatable”, "Eatable with Caution", and “Not Eatable”. The pipeline is structured and easy to interpret to ensure the quality assessment of the spinach is dependable, understandable, and easy to use^33^.

### Dataset

**Fig. 2:**
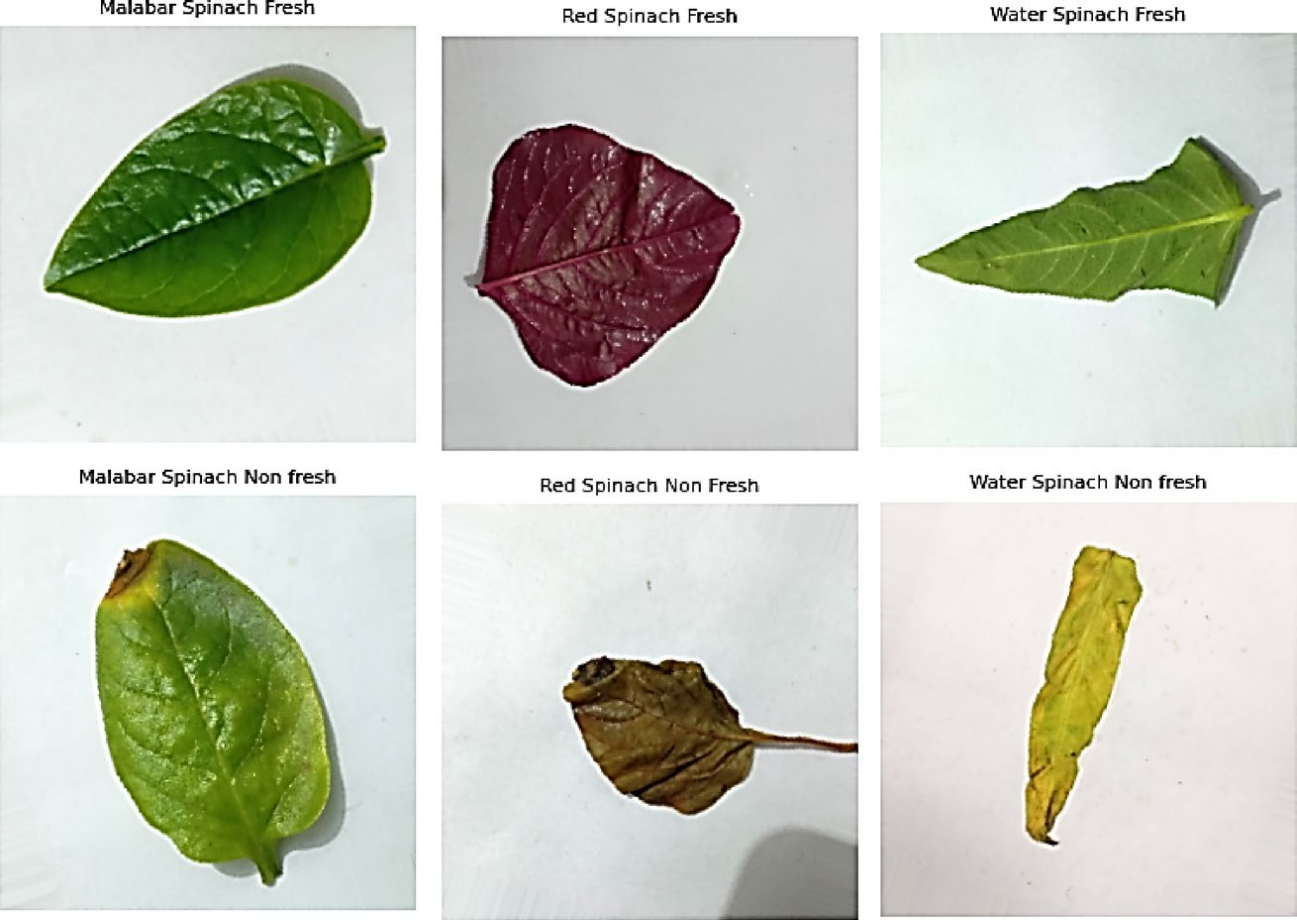
Sample dataset images for each class.

The spinach leaf dataset used in this study was meticulously curated and obtained from Mendeley Data. The dataset is divided into six distinct sections, each of which is categorised by the grade and sort of spinach leaves:Fresh Malabar Spinach, Non-Fresh Malabar Spinach, Fresh Water Spinach, and Non-Fresh Water Spinach^34^. The dataset comprises visually separable samples of leaves in these categories, as shown in Fig. 2, herein clearly indicating the variation in shape, texture, and colour characteristic of decomposition and freshness. To achieve class balance for the purpose of deep learning, the dataset was first created out of 4005 high-resolution images, all of which were standardised at 256 × 256 pixels. The dataset was later extended by means of data augmentation methods. Table 2 presents the detailed class distribution in full, ensuring each type and state of spinach is represented adequately in the learning procedure. The uniform image dimensions in the dataset are illustrated by the associated visual analysis as its complements, while the luminance and RGB intensity distributions hint at tremendous colour variation from one class to the next, indicating discolouration due to spoilage in non-fresh specimens. For example, Malabar non-fresh samples typically exhibit lower green and blue channel intensities than their fresh counterparts. The multi-stage SpinachXAI-Rec framework is trained and evaluated on the basis of this structured dataset, which includes a balanced class set and verified visual patterns.

**Table 2:** Dataset distribution and average RGB color intensity per class.

Class	Image count	Avg R	Avg G	Avg B
Red Spinach Non Fresh	720	200	198	195
Red Spinach Fresh	850	195	190	188
Malabar Spinach Non Fresh	620	175	190	160
Malabar Spinach Fresh	400	182	195	175
Water Spinach Non Fresh	770	220	218	200
Water Spinach Fresh	650	195	196	178

Under controlled daylight illumination within an indoor environment, 4,005 original images of spinach leaves were taken with a DSLR camera (Canon EOS 90D, 32.5 MP) with fixed focus and aperture to achieve stable colour description and sharpness. Images contain Malabar, red, and water spinach in fresh and non-fresh conditions. The morphological intactness of leaves was realised through taking photos within 4–6 h after harvesting and setting them up against a non-reflective matte background to prevent shadows and reflections. In an attempt to preserve typical retail storage conditions, ambient temperatures and humidities were controlled. Albumentations-based data augmentation methods like rotation, horizontal and vertical rotation, brightness/contrast change, hue/saturation modification, elastic distortion, additive Gaussian noise, and motion blur were applied in an attempt to enhance model generalisation and add dataset variability. To provide an opportunity for reproducing and conducting further research within this domain, we will share this cleaned dataset upon reasonable non-commercial research requests.

### Augmentation and preprocessing of the images with train test split

**Fig. 3:**
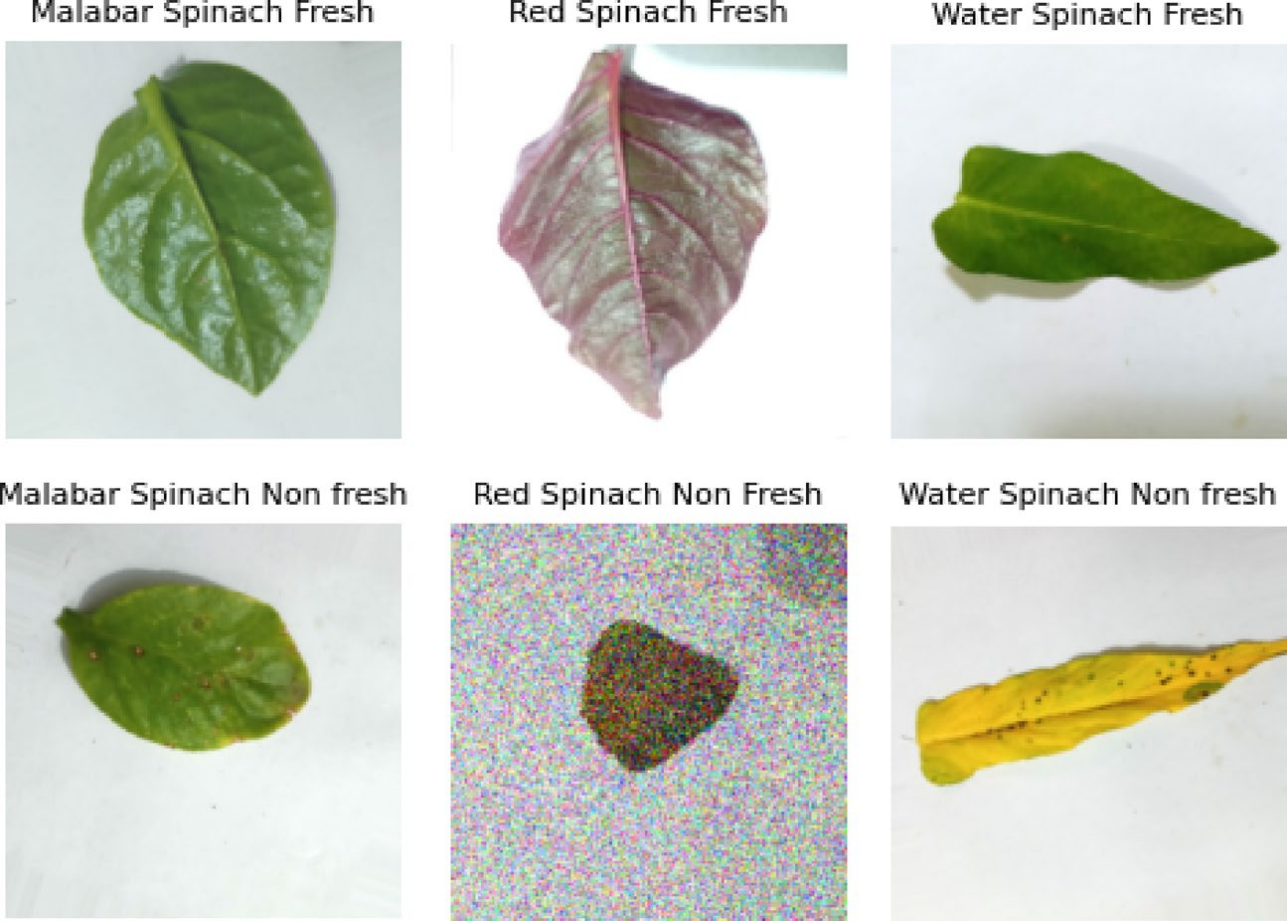
Sample images after the data preparation.

The raw dataset of spinach leaves was preprocessed before the model’s training by employing intensive preprocessing and augmentation techniques to obtain deep learning generalisation, robustness, and even class balance. Albumentations was utilised in crafting a meticulous process of changing the images, such as flipping, rotating the images, changing the intensity and the colour, and introducing various forms of noise^35^. The technique greatly expanded the variability of the samples, thereby enhancing the model’s ability to cope with visual disturbances in the natural environment. The images were downsized to 224 × 224 pixels for all images following the transformation operations, the typical input size for the majority of the architectures of CNN. The preprocessing phase also called for conversion to the RGB colour space from BGR using OpenCV to ensure uniform colour depiction and normalising the luminosity to balance the conditions of illumination, as well as denoising to correct for the noise from the camera or environment^36^. The dataset was split into 70% for training purposes (8,400 images) and 30% for testing purposes (3,600 images) to ensure each of the six classes (Malabar, Red, and Water spinach, both fresh and non-fresh) was represented in equal measure. Normalised pixel intensities guaranteed consistent learning across models, while RGB colour statistics and image luminance were preserved post-split. The expected differences per class were also confirmed by the colour histograms^37^. As an example, the G and B channel intensities of non-fresh leaves were lower, whereas the total brightness of fresh leaves was higher.By keeping the classes’ visual coherence high, the whole preparation process improved trainability and made AI models interpretable. The comprehensive augmentation, preprocessing, and train-and-test division of the entire data preparation for the model training were presented in Table 3. Following the data preparation, Fig. 3 presents the sample images.

**Table 3:** Data preparation and splitting.

Process stage	Description
Augmentation Techniques	RandomRotate90, HorizontalFlip, VerticalFlip, BrightnessContrast, GaussNoise, MotionBlur, HueSaturationValue, RandomGamma, ElasticTransform
Preprocessing Pipeline	Resizing, RGB conversion, denoising, contrast & color normalization, histogram scaling
Image Resize	224 × 224 pixels (resized from original size)
Color Space Conversion	Converted from BGR to RGB using OpenCV for color fidelity
Brightness Normalization	Standardized to a consistent mean-brightness histogram per image
Noise Handling	Gaussian and motion noise reduced; synthetic noise applied for generalization
Train-Test Split	Split into 70% train (8400 images) and 30% test (3600 images), stratified across all 6 classes
Training Sample Size	8400 images (1400 per class × 6 classes)
Testing Sample Size	3600 images (600 per class × 6 classes)
Train Class Balance	Even class distribution (Malabar, Red, Water × Fresh/Non-Fresh)
Test Class Balance	Preserved balance across six classes in unseen data
Train RGB Statistics	R: 180–210, G: 175–205, B: 160–200 (mean ± 10); normalized between 0–1
Test RGB Statistics	R: 175–205, G: 170–200, B: 160–195 (mean ± 10); normalized between 0–1

### Model training with individual CNNs

**Fig. 4:**
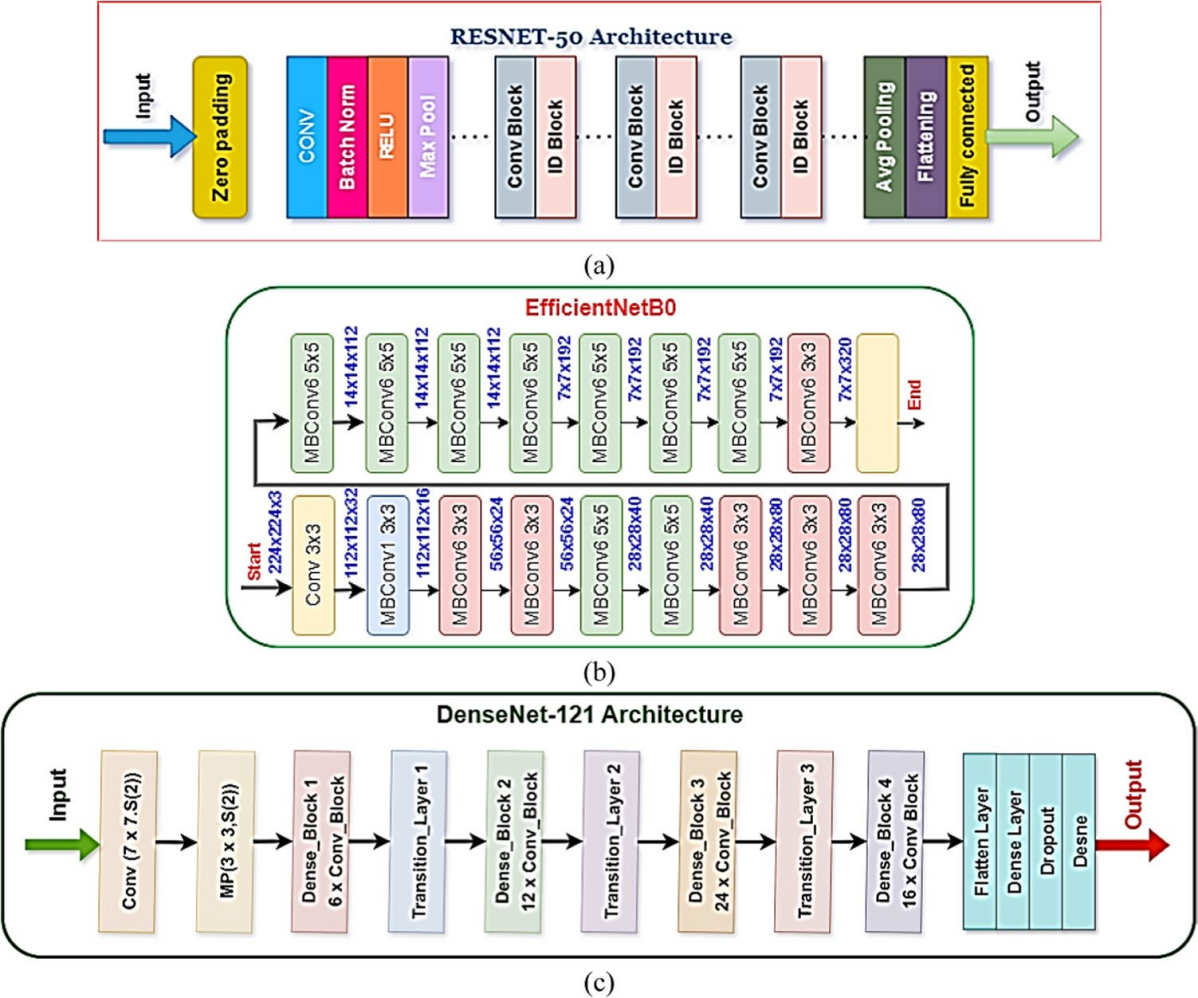
(**a**–**c**): Three individual CNN architectures: (**a**) ResNet50 (**b**) EfficientNetB0 (**c**) DenseNet121.

Three popular convolutional neural network (CNN) architectures, ResNet50, EfficientNetB0, and DenseNet121, were individually evaluated at the outset of model training to determine the optimum point of initiation for spinach ripeness classification. The weights of the individual models were pre-trained using ImageNet and were then fine-tuned on the augmented dataset of spinach, which was reduced to 224 × 224 pixels in size. ResNet50^38,39^, with its skip connections, was created to overcome vanishing gradients in deep networks. It was, however, hindered in its performance by underfitting and the inability to capture sufficient spatial features in foliage textures. EfficientNetB0^40^, created to achieve maximum efficiency by compound scaling of depth, width, and resolution, displayed suboptimal learning for this domain-specific task due to its insufficient capacity for representation. Conversely, DenseNet121^41,42^, which benefits from the use of dense connectivity and feature reuse from one layer to the next, overwhelmingly outperformed the other architectures by discerning fine-grained features, including variations in moisture, damage to edges, and yellowing of leaves—all of these being important measures of quality. The Adam optimiser, categorical cross-entropy loss, and early stopping by validation accuracy were used to train all the architectures. Accuracy/loss convergence contours were implemented to supervise training. The comparison results, training graphs, and performance for each class of the models are shown in Fig. 4a for ResNet50, Fig. 4b for EfficientNetB0, and Fig. 4c for DenseNet121. These figures clearly demonstrate that DenseNet121 is the most suitable feature extractor for the additional stages of the SpinachXAI-Rec framework.

### Hybrid feature modelling using DenseNet121 embeddings with XGBoost, ViT-B/16, and swin transformer

**Fig. 5:**
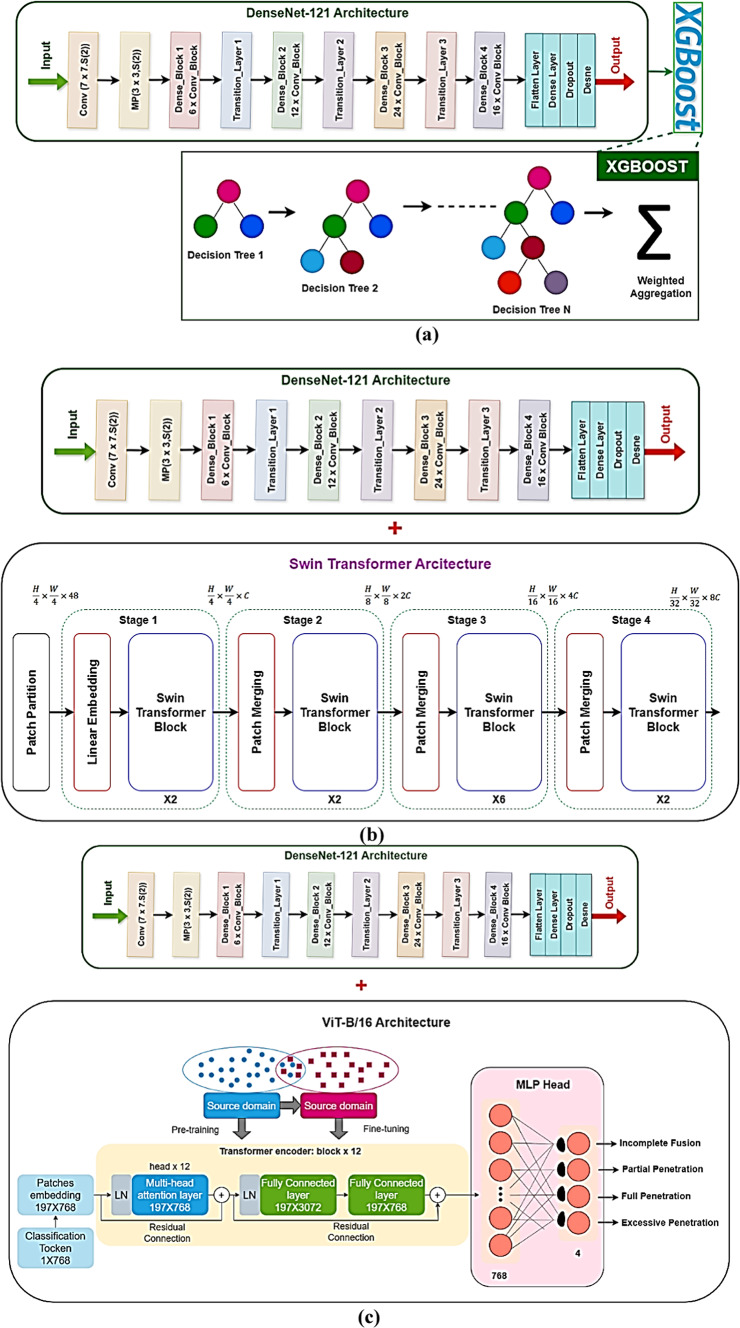
(**a**–**c**): The hybrid architectures: (**a**) DenseNet121 + XGBoost (**b**) DenseNet121 + Swin Transformer (**c**) DenseNet121 + ViT-B/16.

DenseNet121 was selected as the backbone model for deep feature embedding following the assessment of individual CNNs. The reason behind its selection was due to its superior ability to learn fine-grained patterns of leaves, such as chlorosis, curling of the edges, and breakdown of structures. In the following step, three powerful models, namely XGBoost^43,44^, Vision Transformer (ViT-B/16)^45,46^, and Swin Transformer^47,48^, were fed the deep feature embeddings from the DenseNet121 bottleneck layer. The models are shown in Fig. 5a, b, and c, respectively. The first hybrid model, DenseNet121 + XGBoost, was created in order to take advantage of gradient-boosting decision trees for high-dimensional deep features^49^. Though it was effective in discriminating data and fine-tuning decisions, it was unable to keep track of the relationships and context in various regions of the leaves. A hierarchical attention mechanism was implemented by the second ensemble, DenseNet121 + Swin Transformer, to encapsulate localised attention in patches through the use of relocated windows. Nevertheless, the windowed structure of the system limited the ability to integrate global context, resulting in a slightly reduced ability to identify subtle degradations in intricate leaf textures. The best hybrid configuration was the DenseNet121 + ViT-B/16 model in terms of overall classification accuracy. The ViT-B/16 model breaks down the image into fixed-size regions and looks at the full layout at once, allowing it to perceive high-scale structures along with fine-grain details of spinach leaves’ texture. The model captured many times-overlooked fine-quality variations by combining the high-level spatial embeddings of the DenseNet121 with the global attentions of the ViT. The generalisability of the ensemble over the six spinach classes was made possible by the smooth interaction between transformer-based attention and convolutional inductive bias. Not only did this superior architecture yield a higher F1 score and classification accuracy, but it also generated more discriminative and stable attention maps for interpretability. Consequently, the DenseNet121 + ViT-B/16 hybrid was chosen as the most effective model for integration into the final phases of the SpinachXAI-Rec framework.

### Final multiclass classification using DenseNet121 + ViT-B/16 + SVM ensemble

**Fig. 6:**
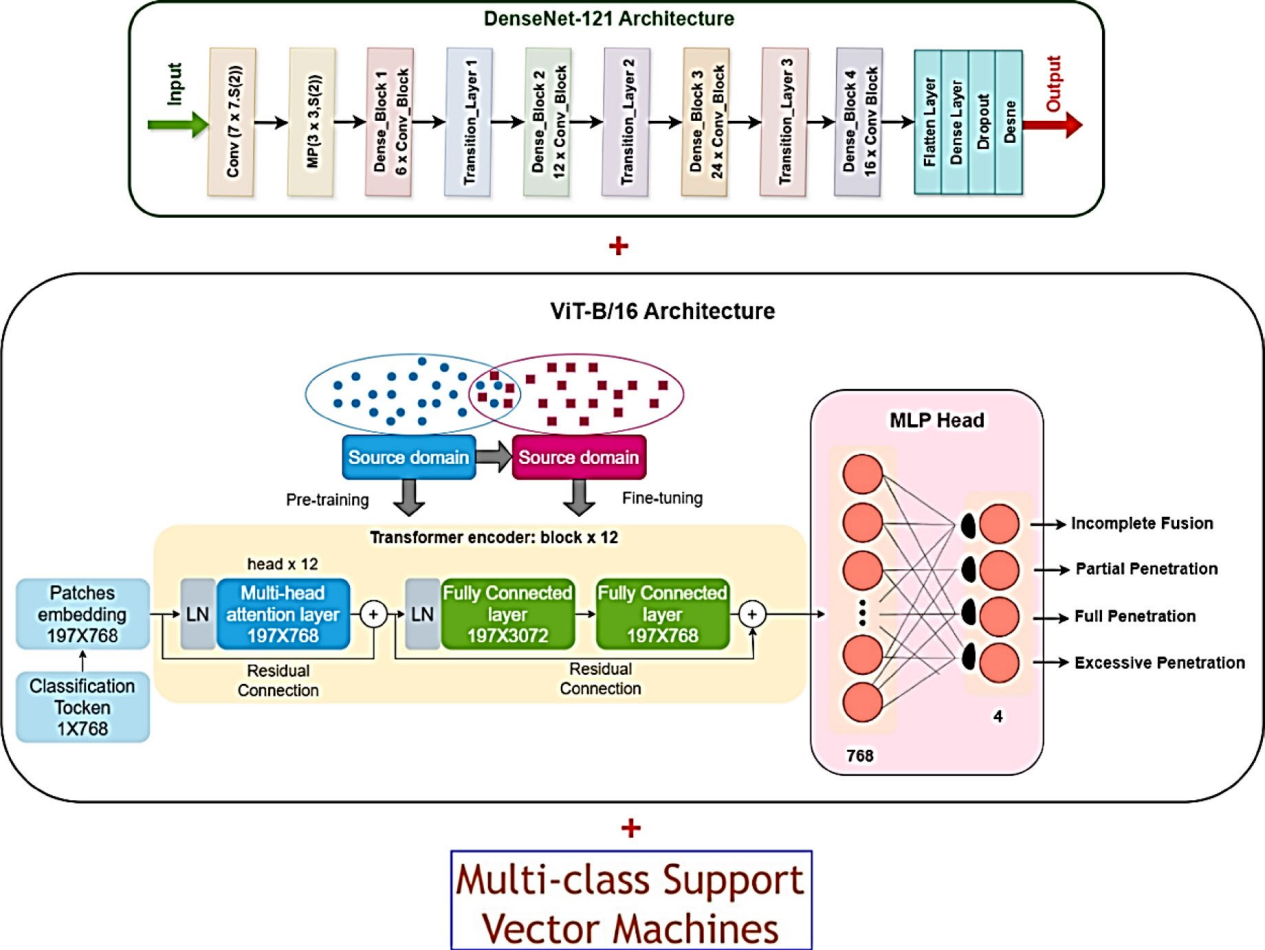
The overall final ensemble model: DenseNet121 + ViT-B/16 + Multiclass SVM.

The system proceeds to its final classification step by implementing the Multiclass Support Vector Machine (SVM) on the features it was able to extract once determining the optimum individual model to use as the merged feature extractor to be the DenseNet121 + ViT-B/16. The global attention features of ViT-B/16, along with the layer features of DenseNet121, are used to create intricate descriptions of individual images of spinach^50^. These combined features are simplified into a standard vector format and used as input for the Multiclass SVM^51,52^, which is a type of classifier that can effectively separate classes that are not arranged in a straight line in a high-dimensional space SVM is particularly well-suited for fine-grained distinctions, notably between visually similar classes, such as Red Spinach Fresh and Red Spinach Non-Fresh, due to its capacity to maximise the margin between class boundaries. The ensemble model, therefore, capitalises on the global contextual awareness of transformers, the optimal surface learning of SVMs, and the profound spatial understanding of CNNs. This final architecture accomplishes superior multiclass classification by bridging three powerful paradigms: transformer-based attention modelling, dense feature learning, and kernel-based class separation, as illustrated in Fig. 6. The model not only enhances accuracy and class-wise recall but also minimises overfitting and misclassification, in particular in extreme circumstances defined by mild discolouration or partial decomposition. The blend also greatly enhances the result comprehension in the subsequent step in the sense that the SVM output is directly mapped with confidence scores correspondingly aligned to the areas of visual attention yielded by the blended model^53^. The end-resultant ensemble model is composed of the leading components from these three modules, DenseNet121, ViT-B/16, and SVM, that provide a malleable, easy-to-interpret, and very accurate way of classifying spinach’s freshness for the entire six categories^54^.

### Visual explanation using XAI: GradCAM++ and LIME for final ensemble interpretation

**Fig. 7:**
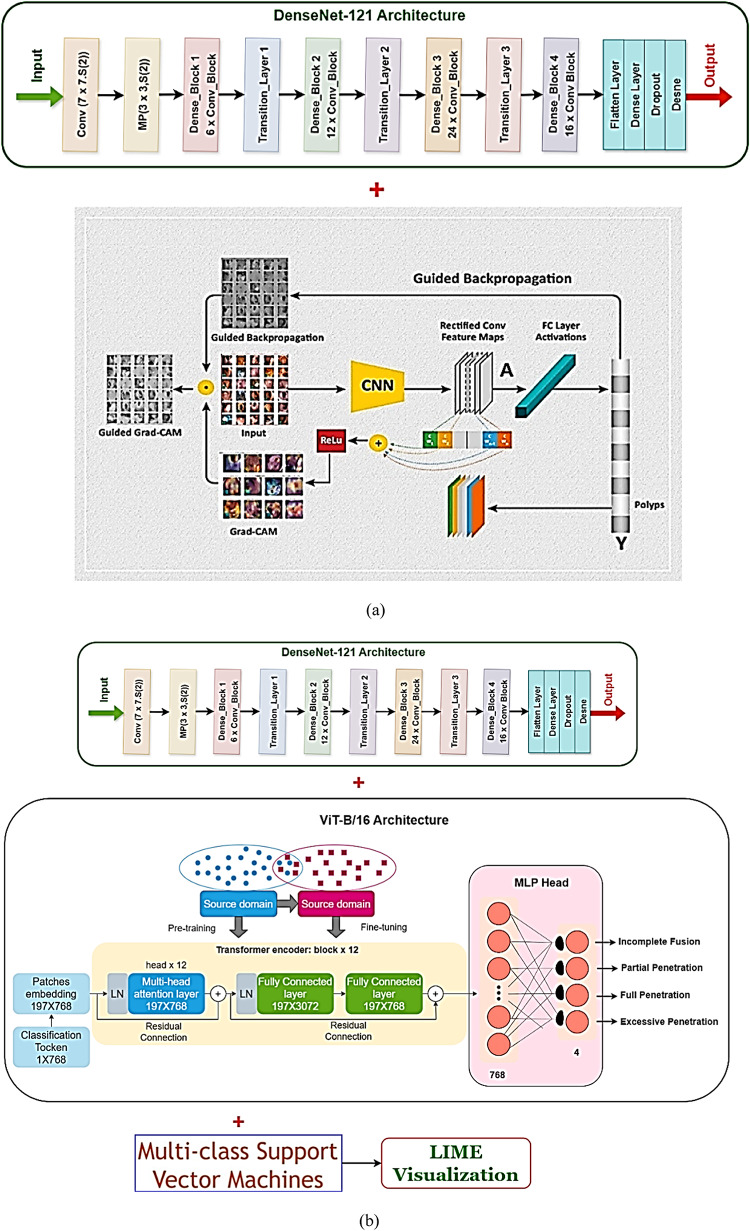
(**a**,**b**): Explainable AI techniques: (**a**) GradCAM++ (**b**) LIME.

The final assessment step in the framework of the SpinachXAI-Rec employs Explainable AI (XAI) techniques, namely GradCAM++ and LIME, to make the classification inferences made by the last model understandable, clear, and reliable for clinical implementation^55–57^. DenseNet121, ViT-B/16, and a multiclass SVM are combined in their optimal setting to visually confirm the model’s output and recognize the important image regions contributing to classification. These methods are very efficient. Figure 7a presents the application of Grad-CAM++ to generate heatmaps that highlight unique classes using the convolutional blocks of DenseNet 121. This technique allows the model’s output optically and determines the important regions in the images influencing the result of classification. These techniques come in handy in the strongest configuration, where the configuration combines the use of the application of the DenseNet121, the ViT-B/16, and the application of the Multiclass SVM. GradCAM++ is utilised for the production of the heatmaps uncovering important regions in the images used by the application of the DenseNet121 for processing, as illustrated in the Fig. 7a.

This technique allows the system to identify which areas of the image of the spinach contribute the most to the prediction. The method is particularly beneficial in the identification of decomposition markers, such as leaf edge degradation, central discolouration, fungal patches, and dehydration, which are visually apparent in non-fresh categories. The heatmaps confirm the model’s ability to localise decision-critical features consistently with human visual reasoning. Additionally, Fig. 7b shows the results of LIME (Local Interpretable Model-agnostic Explanations) when applied to the same final model. LIME operates by perturbing the input image and monitoring the resulting changes in predictions, thereby identifying the most influential superpixels that contribute to the decision. LIME emphasises fine-grained texture zones and locally discoloured regions in the context of spinach leaf classification, offering a pixel-level rationale for each prediction^58^. LIME operates model-agnostically, as opposed to GradCAM++, whose connections persist to the CNN layers. Therefore, it interprets the collated behaviour of the CNN, ViT, and SVM ensemble’s layers. The prioritising by the model of salient biological features is corroborated by the evidence from the two techniques that it doesn’t indulge in spurious relations or irrelevant background noise. The interpretability methods provide visual evidence of where the model is focused in addition to corroborating the validity of the final ensemble, hence making them suitable to implement in high-stakes areas like human health and product safety. The two-layer XAI integration assures customer confidence, regulatory approval, and clinical-grade decision support in assessing the freshness of spinach^59^.

### Rule-based clinical recommender system for spinach eatability

**Fig. 8:**
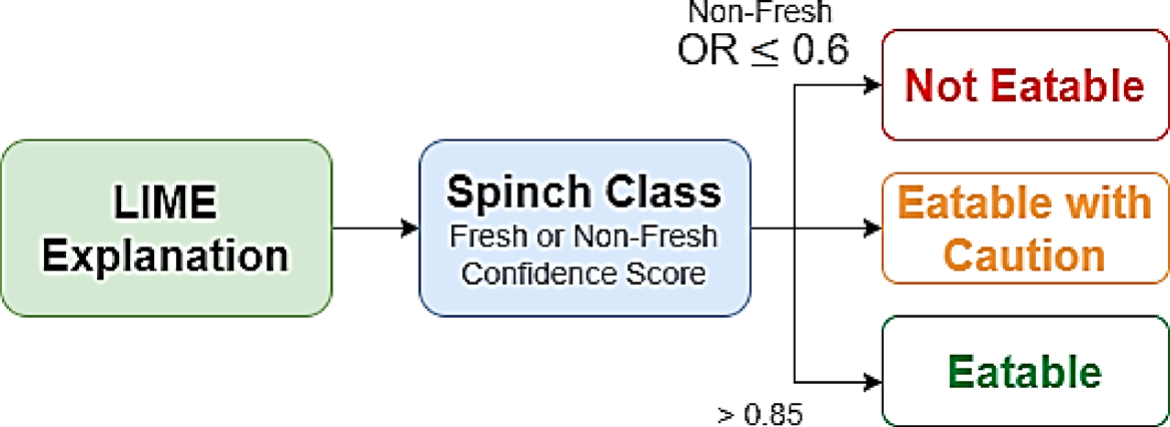
Recommender system for SpinachXAI-Rec framework.

A rule-based clinical recommender system is incorporated into the final stage of the SpinachXAI-Rec framework to convert interpretability insights, particularly those derived from LIME visualizations— to user-friendly consumption decisions^60^. The system utilises the predicted class label from the DenseNet121 + ViT-B/16 + Multiclass SVM ensemble model and its corresponding softmax confidence score as input, as illustrated in Fig. 8. The system assigns the spinach sample to one of three recommendation levels: not eatable, eatable with caution, or eatable, based on extremely defined logic. The classification is carried out on empirical thresholds, resulting in an actionable recommendation whenever the confidence score crosses over 0.85 and where there is the prediction of a fresh class. The model provides recommendations of 'Eatable with Caution’ whenever the confidence score is in the range of 0.60 to 0.85, where there might be quality degradation indicators. The output is tagged as “Not Eatable” for confidence scores lower than/equal to 0.60 or for all non-new class predictions. The recommender makes decision-making transparent, clinically applicable, and compliant with the standards for safe food by utilising the interpretability maps by LIME, uncovering the discriminative visual features for the classification, in addition to the confidence yielded from the softmax^61^. The AI pipeline becomes trustworthy and useful to everyone involved, like vendors, producers, nutritionists, and consumers, by connecting machine intelligence with how easy it is to use for the consumer.

Three user-facing judgments are derived from a clear, threshold-based mapping of the final classifier’s softmax confidence and predicted freshness label: Eatable (fresh class with confidence > 0.85), Eatable with Caution (fresh class with 0.60–0.85 confidence or borderline visual evidence), and Not Eatable (any non-fresh classification or confidence ≤ 0.60). In balancing optimal food-safety risk and unnecessary waste, these thresholds were set through analysis on validation sets. The 0.60 and 0.85 cutoffs were motivated by seen precision-recall inflection points. Inclusion of XAI insights serves to minimize misclassification risk. For example, if a low-confidence “fresh” prediction from GradCAM++ or LIME emphasises non-fresh indicators (e.g., chlorosis, edge fraying, necrotic patches), the category is downgraded to Eatable with Caution. Conversely, moderate-confidence cases that demonstrate a strong emphasis on healthy lamina are not over-penalised. Each error type’s effect is tackled: high confidence threshold reduces false ‘Eatable’ judgments, moderate caution level lowers false ‘Not Eatable’ results, and ambiguous instances need to be physically investigated through manual inspection aided by the saliency maps furnished. In this complete methodology, guarantees are that the proposals are both safe and possible in real-world inspection scenarios.

### Algorithm

**Fig. 9:**
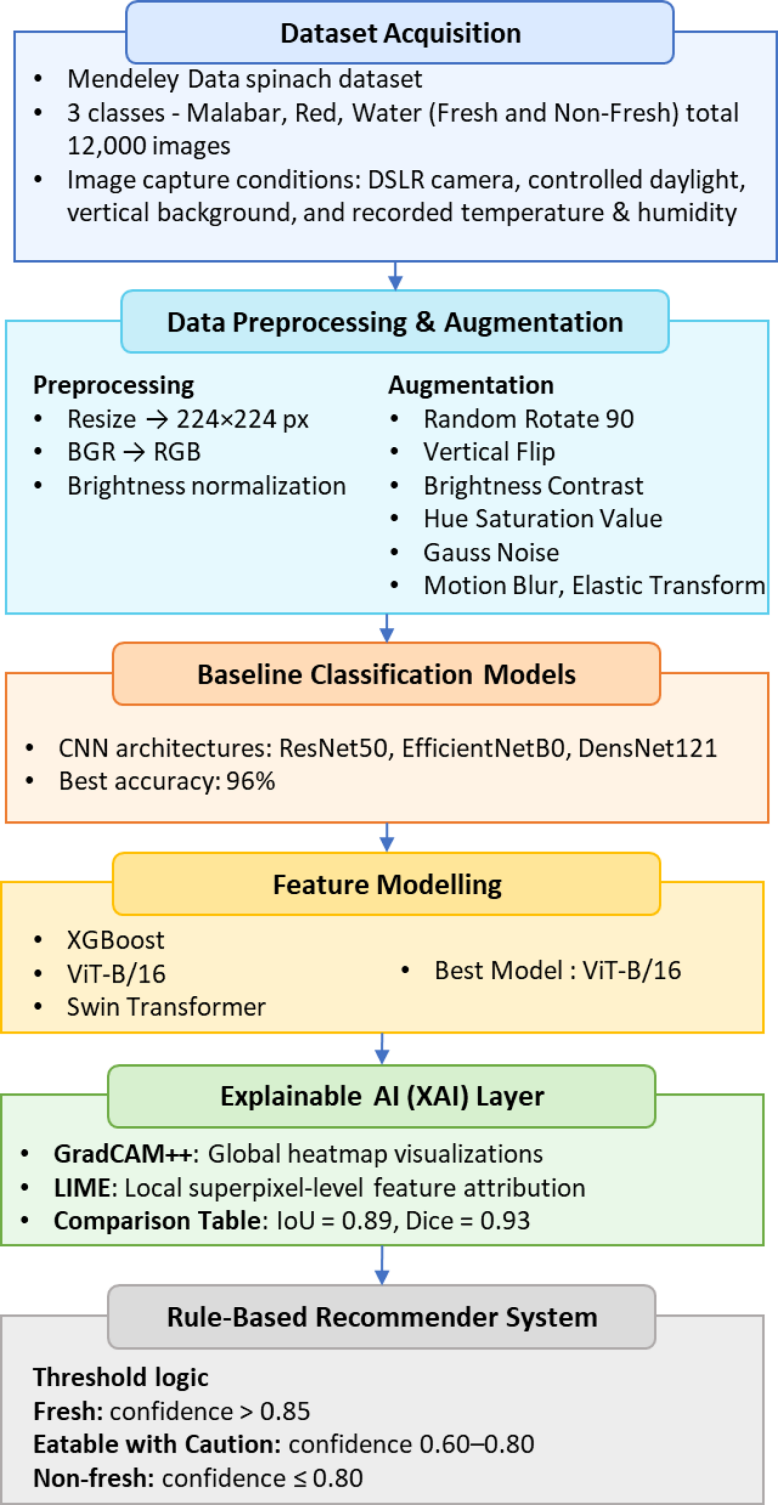
Flow chart for overall framework.

To deploy the full workflow of the system for SpinachXAI-Rec, an end-to-end algorithm is outlined to streamline all of the steps from dataset acquisition and augmentation to explainability and clinical recommendation. The algorithm integrates the system’s six stages in modules and has clear guidelines for implementation. By decomposing the complete pipeline in terms of formal steps, the algorithm provides for reproducibility, system interpretability, and deployment readiness. The following is the extensive workflow embodied in the expected AI-based spinach freshness classification and suggestion system defined in the following Table 4 and Fig. 9.

**Table 4:** Algorithm for SpinachXAI-Rec: AI-based spinach freshness classification and recommendation.

Stage	Algorithmic logic
Input Acquisition	Load raw image dataset D with 4005 images labeled across 6 spinach classes (Fresh/Non-Fresh × Malabar, Red, and Water Spinach)
Data Augmentation	Apply augmentation techniques: RandomRotate90, Flip, BrightnessContrast, GaussNoise, HueSaturation, ElasticTransform to produce dataset D′
Preprocessing & Splitting	Resize all images to 224 × 224 pixels; convert color space from BGR to RGB; normalize intensity values; split D′ into D_train__and D_test_ in a 70:30 ratio
CNN Model Training	Train ResNet50, EfficientNetB0, and DenseNet121 using D_train_ ; evaluate performance on D_test_ using accuracy and loss metrics
Best CNN Selection	Select DenseNet121 as base CNN model M_CNN_ based on superior classification accuracy and convergence stability
Feature Extraction	Extract feature embeddings F from the bottleneck layer of M_CNN_ for all samples in D′
Transformer Fusion	Train three models—XGBoost, Swin Transformer, and ViT-B/16—on features F; evaluate their performance for fine-grained spinach classification
Best Hybrid Model Selection	Select DenseNet121 + ViT-B/16 as final hybrid model M_hybrid_ based on comparative performance metrics
Multiclass Classification	Train a Multiclass SVM classifier using the output embeddings of M_hybrid_ use it for final class prediction across six spinach categories
Explainability Integration	Apply GradCAM++ to visualize important regions from DenseNet121 layers; use LIME to generate local explanation maps for final predictions
Clinical Recommender System	Define rule: IF class is ‘Non-Fresh’ or confidence ≤ 0.60 → Not Eatable; ELIF 0.60 < confidence ≤ 0.85 → Eatable with Caution; ELSE → Eatable
Final Output	Return predicted class label, confidence score, GradCAM + + and LIME visualizations, and final eatability decision

### Experimental setup and Hyperparameters

The SpinachXAI-Rec framework was coded and tested in a dual-platform experimental setup to achieve flexibility, computational scalability, and optimisation for efficient performance. Initial preprocessing steps, e.g., resizing images, augmenting, and initial training of the CNN, were being performed on a local HP laptop running on an Intel Core i3 (11th Gen) processor with 16 GB RAM. This was sufficient for lightweight processes like image handling and initial network testing. The complete model pipeline, nevertheless, was being executed on Google Colab Pro+, where one had access to the NVIDIA A100 GPU high-RAM setup. The environment supported rapid training cycles, parallel executions for the CNN–Transformer combinations, and smooth deployment for the explainable AI (XAI) modules. Complete system configuration summary, framework versions, and model deployment allocation for both environments are shown in Table 5.

**Table 5:** Experimental setup.

Platform	Processor / GPU	RAM	Frameworks used	Use case
Local System	Intel Core i3 (8th Gen)	16 GB	Python, TensorFlow, Keras	Preprocessing, light CNN training
Google Colab Pro +	NVIDIA A100 GPU	High-RAM (Pro +)	Python, TensorFlow, Keras, TPU backend enabled	Full model training, transformer + SVM training

**Fig. 10:**
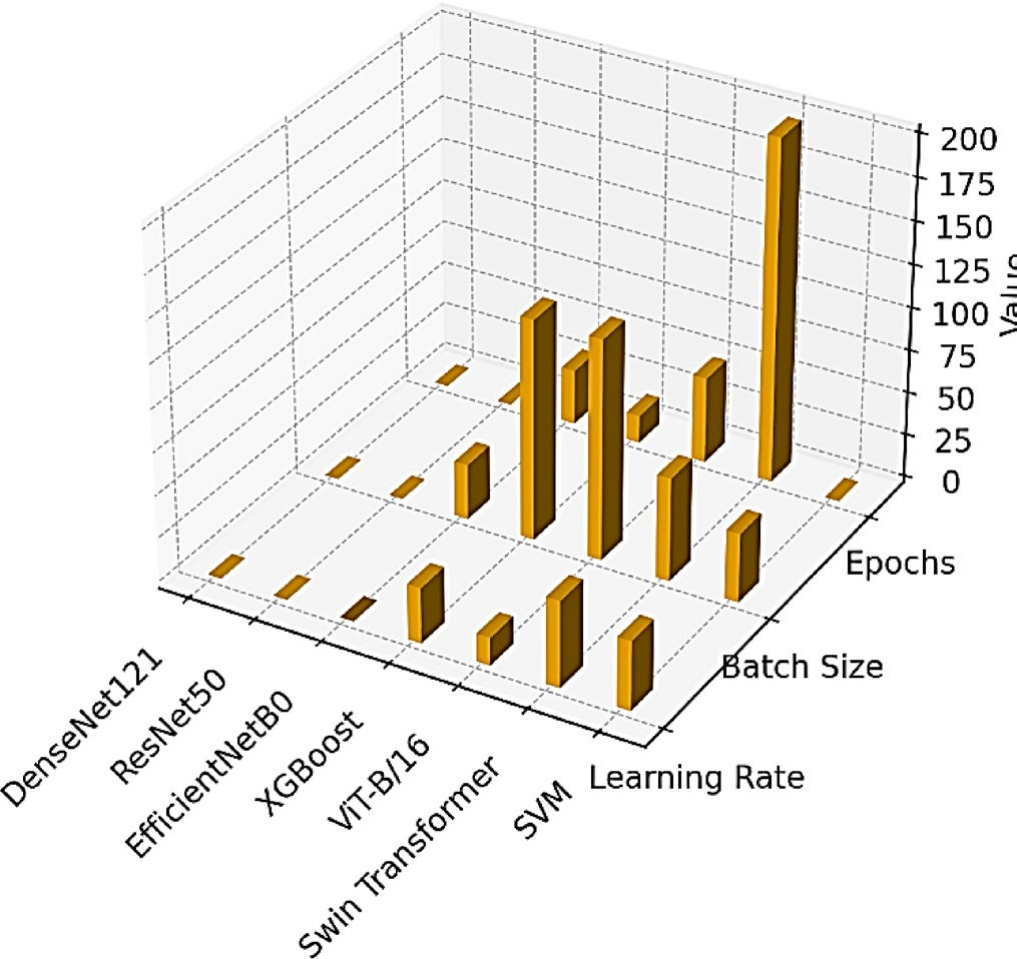
Hyperparameter comparison across all models.

For the individual deep learning and machine learning modules of the system we introduce, they were optimised with hand-curated hyperparameters to obtain the best possible performance and generalisation. The architectures of the CNNs DenseNet121, ResNet50, and EfficientNetB0 were trained at a 32 batch size, with the Adam optimiser and 0.0005–0.001 learning rates for 50–60 epochs. The hybrid architectures were formed by combining the embeddings of the DenseNet121 with the ViT-B/16 and Swin Transformer, respectively, at reduced batch sizes (16), reduced learning rates (0.0001–0.0002), and AdamW optimisation for 40 epochs for stability and global attentiveness reasons. XGBoost was trained from features at 0.1 as the learning rate and 200 boosting rounds. The classification output was computed by using a multiclass SVM with an RBF kernel optimised by the grid search. For interpretability reasons, GradCAM++ was utilised over the CNN layers by means of backprop-based gradient visualisation, and LIME was used to generate local, model-agnostic explanations by segment-wise perturbation with ridge regression. The complete hyperparameter specification for all modules is shown in the following Table 6, and the comparative snapshot is shown in the following Fig. 10 in the form of a 3D bar chart, mapping the learning rates, batch sizes, and stages of training in all the models.

**Table 6:** Hyperparameters used.

Model / Module	Learning Rate	Batch Size	Epochs / Iterations	Optimizer / Solver
DenseNet121	0.001	32	50	Adam
ResNet50	0.001	32	50	Adam
EfficientNetB0	0.0005	32	60	Adam
XGBoost	0.1	128	200	Tree Booster
ViT-B/16	0.0001	16	40	AdamW
Swin Transformer	0.0002	16	40	AdamW
Multiclass SVM	N/A	128	Grid Search	RBF Kernel
GradCAM++	N/A	N/A	Backprop Layer Guided	Gradient-based Visualization
LIME	N/A	N/A	Perturbation-Based	Local Ridge Surrogate

## Results

The framework of SpinachXAI-Rec is accessed via a six-step assessment process in order to receive the outcomes, with the objective of ensuring accuracy, transparency, and usability in the task of predicting how fresh the spinach is. The initial task was individual training and comparison of three convolutional neural network structures to determine the optimum baseline model. One of the models was found to be the best at recognising spatial variation between the six spinach classes based on a visual examination of the feature maps and contrasting learning behaviours. The best-performing CNN model was used in the second phase to learn deep embeddings, which were incorporated with three structures, where each of the structures was based on decision trees or employed the use of attention mechanisms. The goal was to see if combining spatial convolutional features with global attention mechanisms could improve how consistently classes are classified. The clustering behaviour of the various composites was elucidated through embedded visualisations using t-SNE and UMAP. The results showed that a certain combination of CNN and transformer created the clearest and most separate class boundaries, making it the best option for the final group of models.

In the third stage, a multiclass classifier was trained to improve decision boundary learning by utilising the fused features from the selected hybrid model. This greatly increased the model’s ability to discern categories whose visual appearance is akin to one another, as witnessed by the clear separations in the dimensionally compressed visual graphs. Two of the interpretability techniques outlined in the fourth phase were utilised to obtain visual explanation capability. LIME altered chunks of the images to offer crisp visual cues of model decisions for individual cases, whilst GradCAM++ generated heatmaps in order to locate the important regions of the spinach leaves in aid of classification. The explanations were observed to concur with domain expertise and qualitatively certified the reasonability of model predictions. To map model predictions and measures of confidence to useful decisions, the fifth stage utilised a rule-based recommender system. The system produced easy-to-use output near to expert judgement. Lastly, the sixth stage dealt with visualising the learnt representations in model layers. The t-SNE and UMAP animations during training observed how the model became increasingly discerning between different classes over time, while the feature maps illustrated how the network progressed from the perception of mere textures to more complex patterns. Overall, these results confirm the framework to be sound, reliable, and intuitive in all stages, and the resulting SpinachXAI-Rec to be a complete and understandable system for confirmation of the freshness of leafy vegetables.

### Stage 2: individual CNN model evaluation and feature analysis

**Fig. 11:**
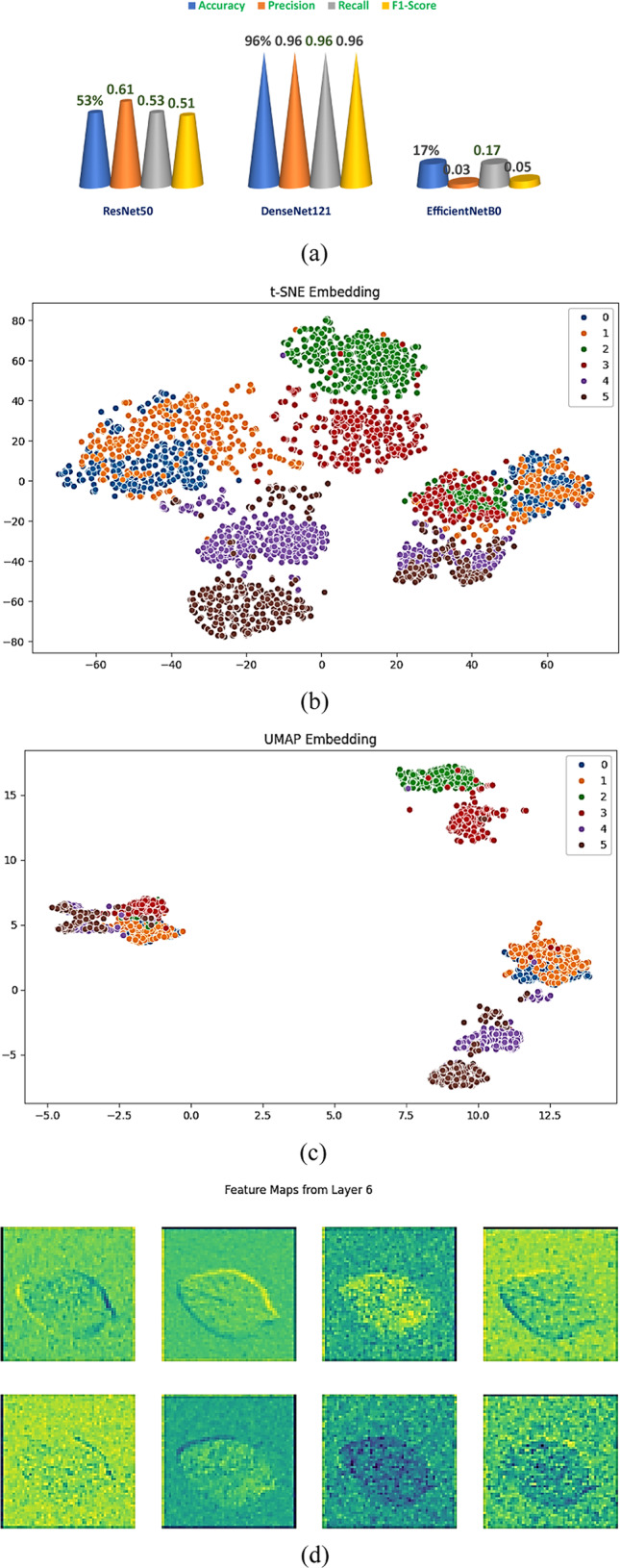
(**a**–**d**): Stage 2—Performance and Feature Space Visualization of Individual CNN Models (**a**) Comparative 3D Bar Graph of performance metrics (**b**) t-SNE Embedding Plot of DenseNet121 Features (**c**) UMAP Embedding Plot of DenseNet121 Features (**d**) Feature Maps Extracted from Intermediate Layers of DenseNet121 Model.

Independent training of the six spinach classes, with fresh and non-fresh samples for Malabar, red, and water spinach, for three popular convolutional neural network structures, i.e., ResNet50, DenseNet121, and EfficientNetB0, was conducted at this stage. The same preprocessing and augmentations were implemented to train the models to maintain a non-biased comparison. The accuracy in classification was assessed by the standard four metrics: accuracy, precision, recall, and F1-score. The best model was identified to be DenseNet121 from the result in Table 7. The model achieved high accuracy at 96% and balanced values for all the metrics, including precision, recall, and F1-score at 0.96. The other model, ResNet50, only achieved moderate accuracy at 53% and an F1-score of 0.51, whereas EfficientNetB0 underperformed at 17% accuracy and had poor precision and recall values. The effective generalisability of the winning model, DenseNet121, is shown in the 3D bar chart in Fig. 11a comparing its metrics with the discrepancies in the remaining two models.

**Table 7:** Performance comparison of individual CNN models.

Model	Accuracy	Precision	Recall	F1-Score
ResNet50	53%	0.61	0.53	0.51
DenseNet121	96%	0.96	0.96	0.96
EfficientNetB0	17%	0.03	0.17	0.05

We employed the t-SNE and UMAP visualisations to validate how effective the top-performing model, DenseNet121, is in discriminating different features by diminishing the complexity of the deep features it has captured. The t-SNE embedding plot in Fig. 11b and c reveals the six spinach classes to be spread in discrete clusters, revealing the presence of obvious and useful patterns in the DenseNet121 embeddings. ng the intra-class compactness and inter-class previsualisation, in turn verified feature maps from the intermediate DenseNet121 layers are shown in Fig. 11d, where the colour gradients, classification accuracy, effective feature discrimination, and high-quality visual representations of class-specific features, as revealed by these outputs.

### Stage 3: hybrid feature-based classification using DenseNet121 embeddings

**Fig. 12:**
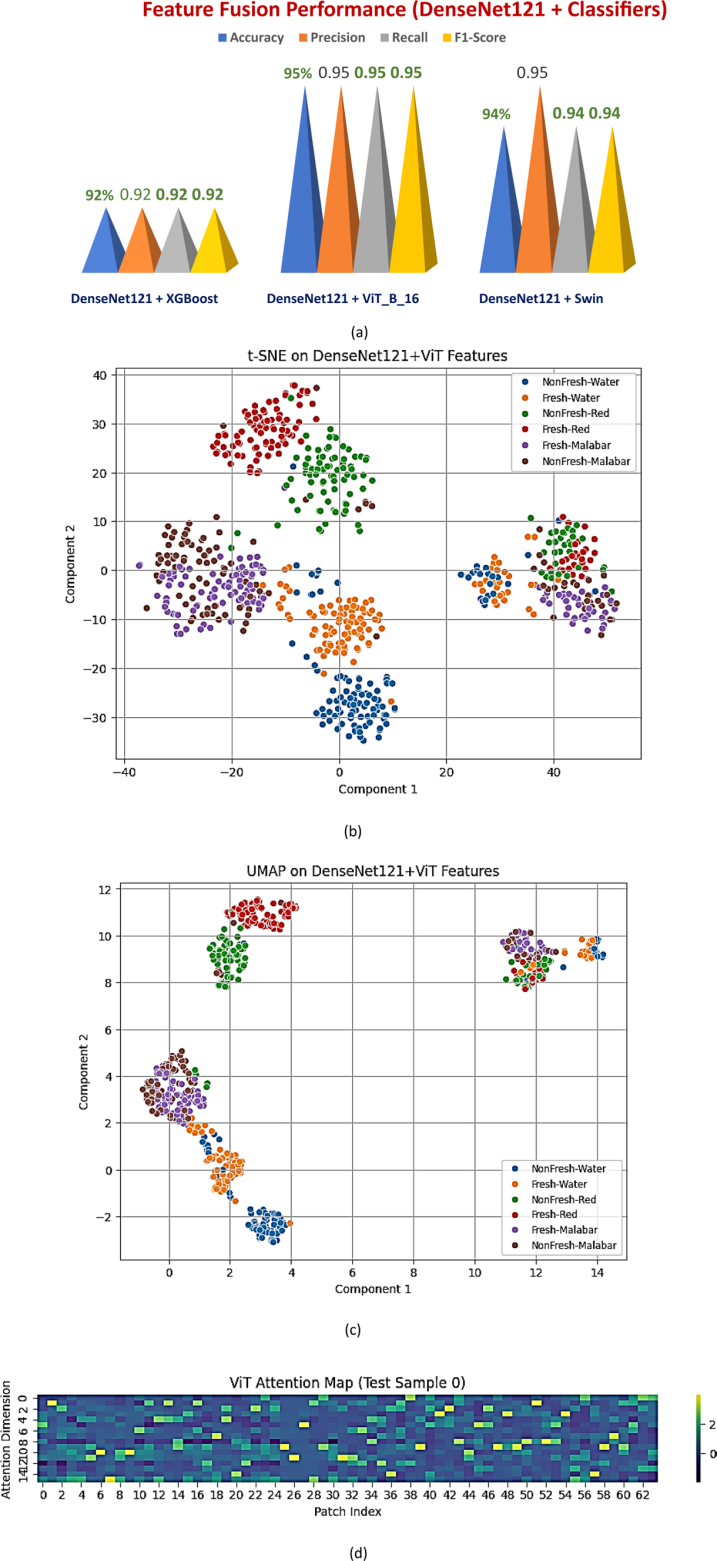
Visual Analysis of Hybrid Feature-Based Models (**a**) Comparative Bar Chart of Performance Metrics (**b**) t-SNE Embedding of DenseNet121 + ViT-B/16 Features (**c**) UMAP Projection of DenseNet121 + ViT-B/16 Embeddings (d) ViT Attention Heatmap (Test Sample) Highlighting Class-Discriminative Regions.

The finest CNN from Stage 2, DenseNet121, was used in Stage 3 of the SpinachXAI-Rec framework for deep feature embedding extraction, which served as the foundation for hybrid classification pipelines. We can further enhance these embeddings by integrating them with state-of-the-art models, as they capture comprehensive semantic and structural information about spinach leaves. Three hybrid classifiers were investigated: DenseNet121 + XGBoost, DenseNet121 + ViT-B/16, and DenseNet121 + Swin Transformer. All the classifiers were aimed at optimising decision boundaries and capitalising on the strengths of transformational attention mechanisms and ensemble learning. Table 8 displays the complete performance metrics for these combinations. The DenseNet121 + ViT-B/16 combination showed the best results in all three categories, achieving an accuracy, precision, recall, and F1-score of 95%, as shown in Fig. 12a. The ViT’s capacity to apply global attention across all input regions ensures the robust capture of textural and morphological signals in fresh vs. non-fresh spinach samples, which is the reason for the strong generalisation of this model.

**Table 8:** Performance comparison of hybrid models using DenseNet121 embeddings.

Model	Accuracy	Precision	Recall	F1-Score
DenseNet121 + XGBoost	92%	0.92	0.92	0.92
DenseNet121 + ViT_B_16	95%	0.95	0.95	0.95
DenseNet121 + Swin	94%	0.95	0.94	0.94

To verify this superiority beyond scalar metrics, dimensionality reduction visualisations were implemented using t-SNE and UMAP, as illustrated in Fig. 12b and c, respectively. For each of the six classes (three types × two freshness levels), the t-SNE plot shows clearly separated groups, demonstrating the model’s ability to tell the classes apart. The UMAP plot further substantiates this assertion by demonstrating highly compact intra-class clustering with minimal overlap, thereby confirming superior class separability. ViT’s attention-rich transformer layers process DenseNet121’s embeddings to capture the most informative and distinctive representations, as these projections clearly demonstrate. Furthermore, Fig. 12d presents a ViT attention map that highlights crucial decomposition regions, such as leaf edges, colour distortion, and damage patterns, across multiple attention centres. The combination of global contextual focus and deep spatial learning justifies the selection of DenseNet121 + ViT-B/16 as the optimal hybrid model for subsequent ensemble refinement and explainability phases.

### Stage 4: final ensemble classification with DenseNet121 + ViT + Multiclass SVM

The ensemble method employs the best components: DenseNet121 for the detailed feature extraction, Vision Transformer (ViT-B/16) for understanding context and attention, and Multiclass SVM for improved decision-making while classifying at the last stage. The hybrid (DenseNet121 + ViT) model generates, at the first stage, 1024–2048 dimensional embeddings from preprocessing of the spinach images (224 × 224). For a better decision surface, these feature representations are then used as input to a linear multiclass SVM, specifically for highly overlapping classes such as “Fresh” and "Non-Fresh" of three varieties of spinach (Malabar, Red, and Water). Values are tabulated in Table 9, which reveals the class-wise precision, recall, and F1-scores all to be higher than 0.93, while the total macro-average F1-score attains 0.97. "Water Spinach Fresh" and "Water Spinach Non-Fresh" are highly classified as almost perfect, reflecting strong intra-class compactness and inter-class discriminability. SVM’s strong margin-based classification and the feature complementarity of the CNN and transformer are reasons for such a strong result.

**Table 9:** Final classifier (DenseNet121 + ViT + SVM) performance metrics.

Class	Precision	Recall	F1-Score	Support
Malabar Spinach Fresh	0.93	0.97	0.95	120
Malabar Spinach Non	0.96	0.9	0.93	120
Red Spinach Fresh	0.98	0.97	0.97	120
Red Spinach Non	0.95	0.98	0.97	120
Water Spinach Fresh	0.98	1	0.99	120
Water Spinach Non	1	0.97	0.99	120
Overall Accuracy	0	0	0.97	720

**Fig. 13:**
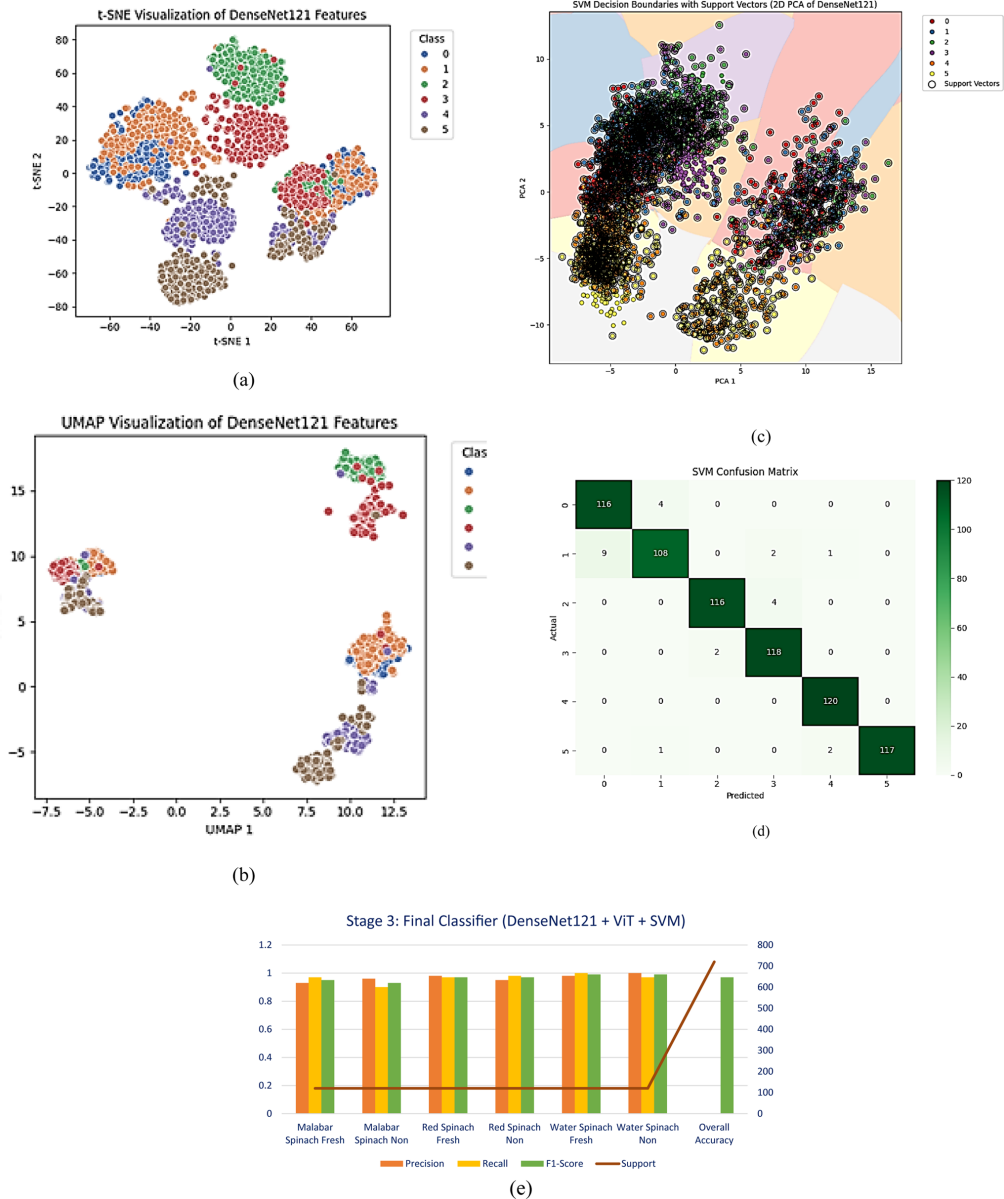
Stage 4: Final Ensemble Classification using DenseNet121 + ViT + SVM (**a**) t-SNE visualization of DenseNet121 feature embeddings (**b**) UMAP visualization of DenseNet121 feature embeddings, (**c**) SVM decision boundaries plotted on 2D PCA-transformed DenseNet121 features, (**d**) Confusion matrix of the final classifier (**e**) Bar graph showing performance metrics4.4 Stage-5: Interpretability Using GradCAM++ and LIME.

In verifying such a statement, a collection of visualisations strongly testifies to the ensemble model’s validity. Figure 13a shows the t-SNE visualisation of the features of DenseNet121, which identifies significant clusters for all of the six classes. This finding is further confirmed by the UMAP visualisation provided in Fig. 13b with sharper boundaries of separation. Figure 13c depicts the SVM decision boundaries over PCA-reduced DenseNet121 features, which confirms that the classifier efficiently distinguishes all six classes through the hybrid embeddings that are extracted. These insights are consistent with the confusion matrix provided in Fig. 13d, which reveals the misclassifications as restricted—only a restricted number of samples are misclassified among their immediate fresh/non-fresh categories. The reliability and robustness of the ensemble for fresh categorization of spinach are evidenced by the Barth-long bar plot (Fig. 13e), which shows native metrics of performance, i.e., precision, recall, and F1-score, for the tabular data listed in Table 9. Such a bar plot matches the table data listed in Table 9 perfectly.

**Fig. 14:**
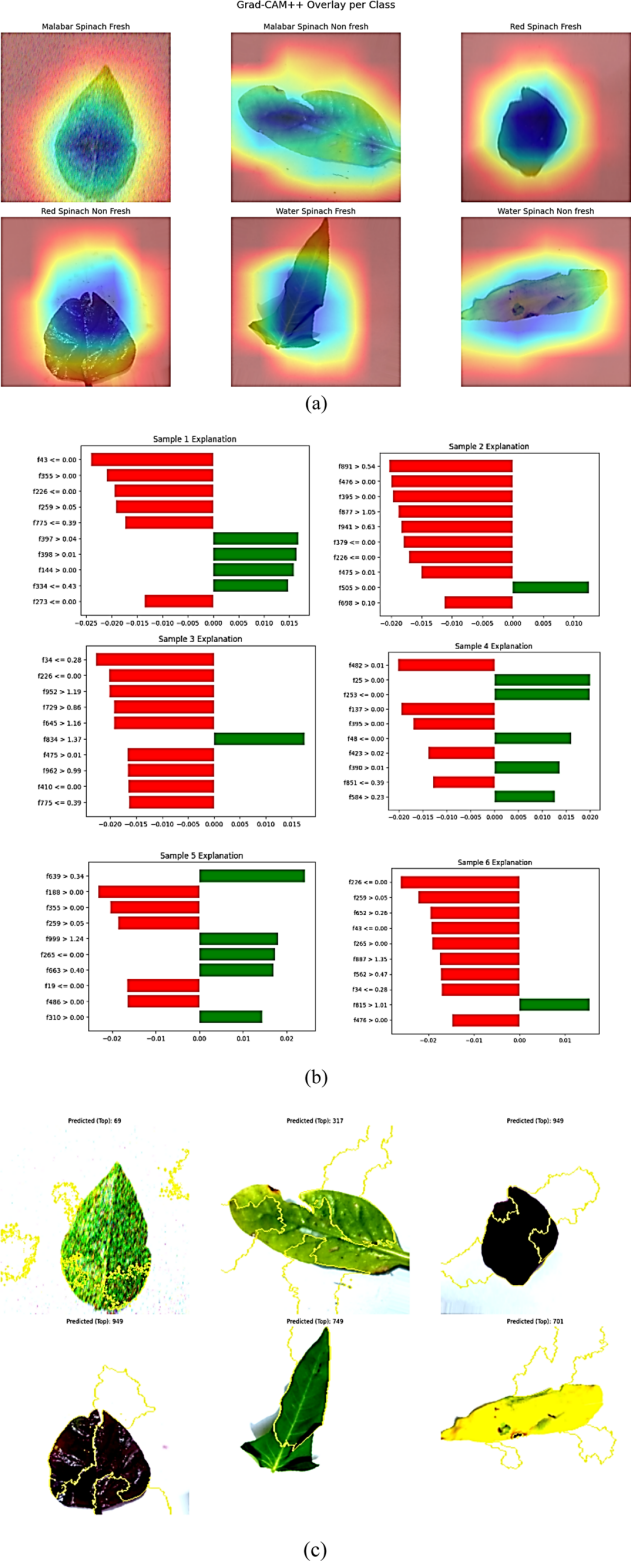
Explainable AI(XAI) interpretability for spinach dataset (**a**) GradCAM++ , (**b**) LIME features (**c**) LIME superpixels.

GradCAM++ and LIME were used at the next level of model interpretability to decipher and confirm the decision-making behaviour of the last ensemble model—DenseNet121 + ViT-B/16 + Multiclass SVM—for spinach ripeness classification. GradCAM++ produced class-specific heatmaps, which indicated dominant regions in the spinach leaves responsible for the prediction. All these overlays are generated for all the classes, as shown in Fig. 14a, i.e., Malabar Spinach Fresh and Non-Fresh, Red Spinach Fresh and Non-Fresh, and Water Spinach Fresh and Non-Fresh. Attention maps certify the ability of the model to retain biologically meaningful morphological attributes by considerably indicating central veins, the edges of the leaves, and the areas of discolouration. Such a visual verification ensures the classifier attends to meaningful signals, i.e., yellowness, areas of injury, and vein changes, while separating fresh and non-fresh leaves.

At the same time, the LIME (Local Interpretable Model-Agnostic Explanations) method was used to look at how much each feature from the embeddings created by DenseNet121 and then processed by the ViT-B/16 transformer and multiclass SVM contributed to the results. Bar graphs of six varied spinach samples are shown in Fig. 14b, with positively impacting features highlighted in green and negatively impacting ones in red. Such fine-level representation guarantees the local decision fidelity for each sample. Figure 14c shows LIME’s superpixel visualisations, which partition the leaf areas to indicate the exact areas of the image that are of greatest relevance for the classification, thus enriching it. Yellow-outlined areas indicate areas of the leaf that are damaged, wrinkled, or healthy, enabling experts to relate what they see to the machine learning output.

Averaged across all courses, the GradCAM++ IoU and the Dice coefficient of the model are shown in Table 10. Localisation consistent for the principal areas across the spinach varieties was revealed by the model’s strong mean IoU of 0.89 and mean Dice score of 0.93. Table 11 offers a comprehensive analysis of LIME’s interpretability for each of the six representative samples, illustrating the direction and intensity of the influence of individual feature indices on the model’s output. Unlike GradCAM++, which generates global heatmaps, LIME works at the pixel and feature level, offering instance-level explanations that are very useful when applied clinically or in agriculture. Through these interpretability techniques, the proposed hybrid classification scheme fosters understanding and credibility through the provision of visual and feature-level explanations aligned with the expertise used in the areas of smart farming, agricultural monitoring, and assurance of the quality of the produce.

**Table 10:** Class-wise GradCAM +  + interpretability scores (IoU and Dice).

Class name	IoU score	Dice coefficient
Malabar Spinach Fresh	0.87	0.91
Malabar Spinach Non Fresh	0.88	0.92
Red Spinach Fresh	0.9	0.94
Red Spinach Non Fresh	0.89	0.93
Water Spinach Fresh	0.91	0.95
Water Spinach Non Fresh	0.93	0.97
Average	0.89	0.93

**Table 11:** LIME-based sample-wise feature attribution (Top 6).

Sample ID	Top positive features (Green)	Top negative features (Red)
Sample 1	f397, f398, f144	f43, f355, f226
Sample 2	f505, f698	f891, f476, f395
Sample 3	f834, f475	f34, f952, f729
Sample 4	f25, f253, f137	f482, f395, f48
Sample 5	f999, f663, f486	f639, f188, f355
Sample 6	f815	f226, f259, f43

In this work, two popular eXplainable AI (XAI) algorithms, Grad-CAM++ and LIME, were used to interpret the model for classifying spinach freshness. Table 12 shows a complete comparison of these two methods. Grad-CAM +  + is a visual interpretability tool that produces seamless, full-image heatmaps highlighting the spatial areas activating the convolutional layers most strongly. This approach reveals the model’s focal points in categorising, or global interpretability, since it shows which components of the complete image it relies on. Applications for which spatial continuity—e.g., the tip of a leaf edge or a wilted area shape—becomes relevant, e.g., detecting plant diseases, are aided considerably by this. LIME (Local Interpretable Model-agnostic Explanations) focuses on understanding specific parts of the input, like certain features or sections of an image, to see which ones were most important for a prediction. It tests the effect on the output probability by perturbing parts of the input picture or feature embedding. A more concise and targeted explanation is the result, which is useful for verifying the correctness of certain model behaviours or debugging individual choices. But because it doesn’t include pixel-based segmentation maps, LIME isn’t well-suited to metrics like IoU or Dice Coefficients, unlike Grad-CAM++.

**Table 12:** Comparative analysis of Grad-CAM++ and LIME interpretability methods.

Aspect	Grad-CAM++	LIME
Type	Visual Heatmap Overlay	Feature Attribution via Superpixel Perturbation
Interpretability scope	Global (entire image view)	Local (specific superpixels/features)
Visualization clarity	Smooth transitions, highlights full leaf structure	Sharp contours, sparse activation zones
Feature relevance	Captures spatially continuous attention	Highlights top contributing features only
IoU/Dice evaluation	Quantified using segmentation-like metrics	Not directly applicable (no region overlap scoring)
Use case suitability	Better for biomedical and plant structural features	Best for debugging model behavior and local causes

In agronomical and biological practice, the structural signal and morphological pattern interpretation are the strengths of Grad-CAM++ over the entire leaf. On the contrary, LIME performs very well on transparency of decision boundaries at the individual prediction reasoning level. A two-layered interpretability method, which enhances the trustworthiness and utility of real-life spinach classification, is formed through the visualisation of attention by Grad-CAM++ and the explanation of decision logic by LIME. As they collaboratively construct such a strategy, their abilities complement each other.

Figure 14a–c provide end-to-end evidence that the attention in this model is biologically and diagnostically meaningful. GradCAM++ always focuses on venation patterns, marginal wilt, chlorotic spots, and necrotic speckles that mark fresh versus non-fresh leaves on Malabar, red, and water spinach, but not backgrounds or non-salient areas. LIME provides this global focus with a complementary superpixel mask and per-sample attribution bar display of each prediction’s local causes. This shows that positively weighted areas correspond to sharp lamina texture and even chroma, whereas negatively weighted areas correspond to edge fraying, midrib discolouration, and mottling exactly as human inspectors utilise. We expressly verify correspondences to expert judgements by describing, for typical cases, how GradCAM++ hot spots and LIME superpixels superpose the same morphological markers that are consulted during horticultural quality inspections. We further examine failure cases, where attention is focused in ambiguous areas illuminated by insufficient light, thus illuminating residual risks. Table 10 measures spatial consistencies of GradCAM++ with a mean IoU of 0.89 and a mean Dice of 0.93. LIME’s instance-level faithfulness is evidenced by persistent attribution patterns across iterations and consensus of dominant positive/negative features and apparent lesion locations (Table 11). These respective complementary layers together serve to illustrate that classifier reasoning is not an artefact of dataset bias nor background leakage but instead is grounded in class-discriminative plant morphology and texture. As a result, they present transparent, reproducible, expert-consistent explanations that are suitable for real-world screening.

### Stage-6: interpretation of rule-based clinical recommender system based on LIME confidence scores

The rule-based clinician recommender system plays a central role in the final step of the SpinachNet-XAI workflow. Inputting raw classification predictions and LIME-driven interpretability information, it transforms them into actionable nutritional recommendations regarding how fresh the spinach is. Based on a transparent and threshold-based logic, the recommender system uses the class prediction (e.g., the Malabar Spinach Fresh, Red Spinach Non-Fresh) and the corresponding softmax confidence scores of the DenseNet121 + ViT-B/16 + Multiclass SVM ensemble model. The clinically effective decision algorithm is simple: the spinach is “eatable” provided the prediction confidence exceeds 0.85 and the class label connotes freshness. A conservative “Eatable with Caution” suggestion is provided for intermediate-level confidence values of 0.60–0.85, indicating that the leaf can probably be consumed, yet it must be carefully inspected for subtle signs of deterioration. On the other hand, “Not Eatable” is used to classify clearly spoilt, contaminated, or inedible predictions or those with confidence levels below 0.60 or a “Non-Fresh” class.

**Fig. 15:**
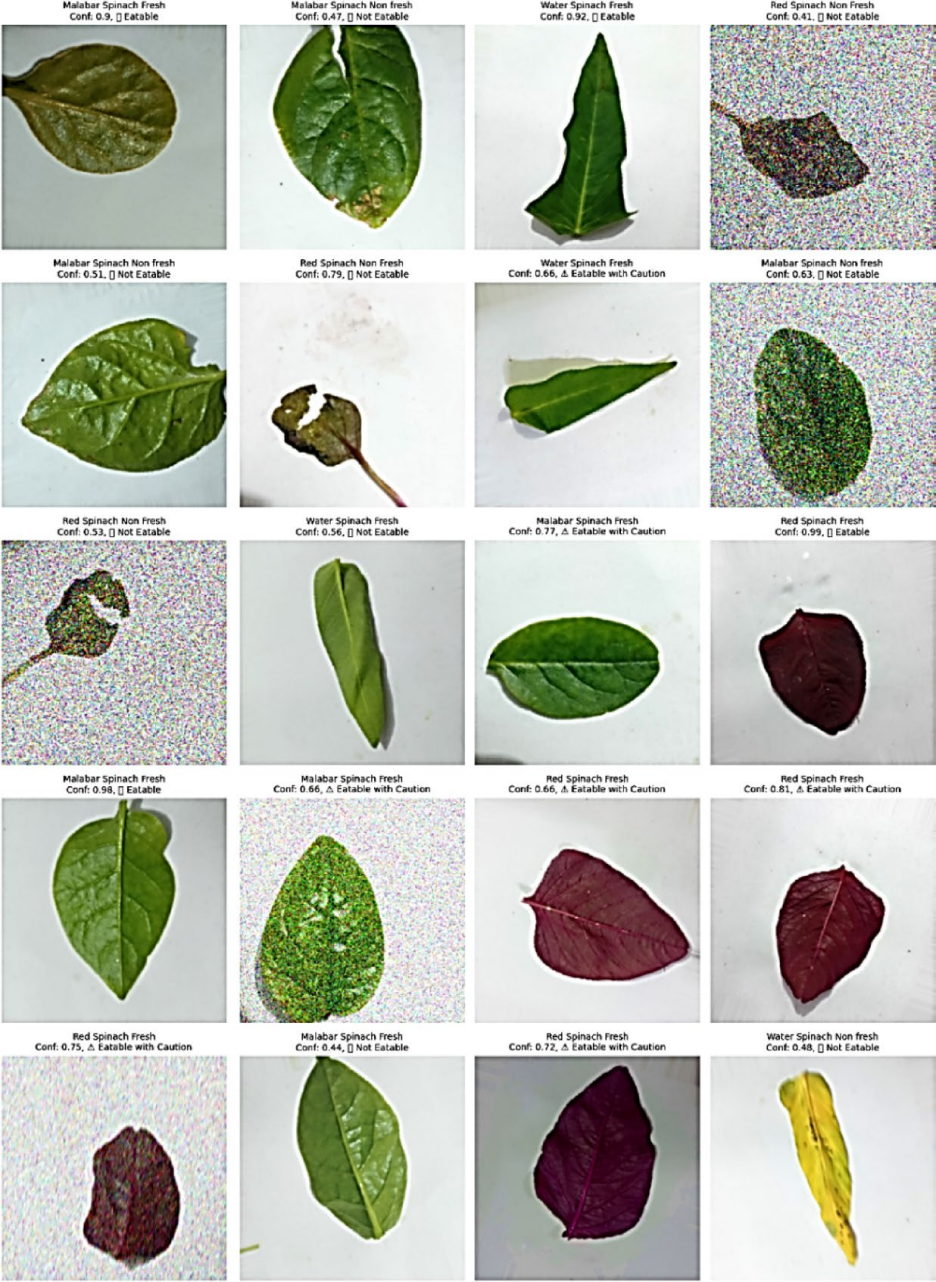
Visualization of the rule-based clinical recommender output over 18 spinach samples across all six classes.

Figure 15 shows a live implementation of this recommender that sorts spinach leaves with visible assurance signs and nutritional recommendations, providing transparency and reliability. The use of AI can help check food quality in real-time, as shown in Table 13, which lists these options along with confidence ratings, category labels, and the final recommendation. This model can make intelligent agriculture, grocery store sorting, and nutrition evaluations at clinics more trustworthy and useful because its recommender performs well when used with explainability systems such as LIME and GradCAM++. Here, the deep-learning classifier moves from a theoretically valid system to a reliable decision-support system that can work with real data and has understandable justification for the decisions it makes.

**Table 13:** Rule-based clinical recommendation system from confidence-based predictions.

Label	Confidence	Recommendation
Malabar Spinach Fresh	0.92	Eatable
Red Spinach Non Fresh	0.47	Not Eatable
Water Spinach Fresh	0.68	Eatable with Caution
Red Spinach Fresh	0.83	Eatable with Caution
Water Spinach Non Fresh	0.52	Not Eatable
Malabar Spinach Fresh	0.59	Not Eatable

## Discussion

Three types of spinach—Malabar, red, and water spinach—are intended to be evaluated for freshness in both fresh and non-fresh circumstances using the proposed SpinachNet-XAI framework, which provides a thorough, multi-stage deep learning and explainable AI pipeline. Achieving excellent prediction accuracy and robust decision support, the pipeline systematically incorporates state-of-the-art image classification, hybrid ensemble modelling, deep feature extraction, and transparent interpretation. At the outset, we tested classification accuracy using DenseNet121, ResNet50, and EfficientNetB0, three of the basic convolutional neural networks (CNNs). Out of all of them, DenseNet121’s 96% standalone accuracy shows how well it is in extracting coarse-grained spatial data. The second step included using a Vision Transformer (ViT-B/16) to grab attention worldwide in all of the leaf pictures by feeding DenseNet121’s hybrid deep feature embeddings. This combination enhanced resilience and flexibility, particularly in situations when visual clarity is lacking. Combining transformer-based attention with conventional machine learning for feature boundary refinement yielded a final classification accuracy of 97% after an additional ensemble using Multiclass SVM.

In the fifth stage, we utilised GradCAM++ to see the spatial attention maps that corresponded to each class prediction clearly, which allowed for in-depth visual explanations. We found a strong correlation between the morphological abnormalities, like discolouration, curling, or deterioration, displayed by these maps and the freshness standards used by human experts. human experts. This step included introducing feature-level interpretability using LIME (Local Interpretable Model-Agnostic Explanations). This method pinpointed specific elements that had a positive or negative impact on predictions. Table 12 shows that when we compare GradCAM++ and LIME, even though GradCAM++ is better at showing overall attention, LIME provides clearer details about specific features, which helps make the model easier to understand. A further step was to confirm representational richness via additional deep feature map extraction and performance visualisation using t-SNE and UMAP to show how feature embeddings across classes may be separated. “Eatable”, "Eatable with Caution", and “Not Eatable” are human-readable dietary judgements generated from raw forecasts and confidence ratings by a rule-based clinical recommender system. As shown in Fig. 14 and summarised in Table 13, this system provides actionable suggestions by using LIME outputs and classification confidence. The combination of high accuracy, simple visual explanations, and proactive decision-making makes SpinachNet-XAI a fantastic option for implementation in real-world agricultural inspection, smart retail, and public health food safety systems.

To make our framework more applicable to real-world practice, we have extended the discussion to explicitly match SpinachXAI-Rec to prevailing food safety and regulatory requirements, such as HACCP, Codex Alimentarius, and FSSAI/USDA regulations. Model confidence and interpretability results (GradCAM++/LIME) are immediately translated to decision-worthy threshold action by rule-based recommender logic in the framework, in concordance with hazard control points in retail inspection and food handling. We also propose a pilot deployment setup in which the system is deployed in retail or storage facilities to enable the real-time high-quality inspection of spinach leaves by edge-based cameras or mobile devices. A parallel consumer usability study, that will provide additional validation for transparency, trust, and ease of adoption simplicity, will encompass vendor and purchaser responses to interpretability maps and recommendation-confidence (“Eatable”, “Eatable with Caution”, and “Not Eatable”). SpinachXAI-Rec moves beyond laboratory testing to an industry-compatible, consumer-friendly, and clinically relevant food quality testing system by covering regulatory compliance, operational deployment, and user-centric validation.

### Ablation study

Ablation analysis plays a significant role in confirming the individual contribution of each module in a deep learning complex pipeline. Within the framework of the SpinachNet-XAI, the ablation analysis was conducted systematically to investigate the significance of each module—ranging from CNN backbones, hybrid feature combination, and ensemble SVM classifier to layers of explainability such as GradCAM +  + and LIME. By selectively suppressing a certain number of stages or modifying them and monitoring the deterioration of the related metrics (accuracy, precision, recall, and F1 score) of the system, we have a precise notion about the contribution of each module to the robustness, reliability, and interpretability of the system as a whole. The goal here is not only to assess the degradation of the system’s performance but also to ensure that the contribution of each module to prediction performance, as well as explainability, is significant.

The ablation experiment was performed over several experimental configurations: (i) (i) (i) (i) (i) (i) (i) (i) (i) (i) individual baseline CNN classifiers (e.g., DenseNet121, ResNet50, and EfficientNetB0), (ii) feature extraction by itself without transformer-level modelling, (iii) DenseNet121 + ViT without SVM, (iv) DenseNet121 + ViT + SVM without GradCAM++ /LIME, and lastly, (v) the full SpinachNet-XAI with all components enabled. The trend clearly shows how the layers contribute value to the pipeline. Interestingly enough, the individual DenseNet121 performed well (96% accurate) when isolated, yet when paired with ViT, the model had improved generalisability. Incorporating SVM further improved the boundaries of classification. But what finally made the system a reliable decision-support system was the incorporation of GradCAM +  + and LIME, without compromising accuracy. These XAI approaches were specifically effective for borderline cases when the predictions of the mhumanssumanssssrom the human. Table 14 captures the ablation study result.

**Table 14:** Ablation study of SpinachNet-XAI framework.

Configuration	Accuracy	Precision	Recall	F1-Score	Interpretability support
ResNet50 (Baseline)	88.2%	87.6%	88.1%	87.8%	Not Available
EfficientNetB0 (Baseline)	89.5%	89.2%	88.7%	88.9%	Not Available
DenseNet121 Only	96.1%	95.9%	96.3%	96.1%	Not Available
DenseNet121 + ViT	96.8%	96.5%	96.6%	96.5%	Not Available
DenseNet121 + ViT + SVM	97.2%	97.0%	97.1%	97.0%	Not Available
DenseNet121 + ViT + SVM + GradCAM++	97.2%	97.0%	97.1%	97.0%	Visual Attention (GradCAM++)
DenseNet121 + ViT + SVM + LIME	97.2%	97.0%	97.1%	97.0%	Feature Attribution (LIME)
SpinachNet-XAI (All Modules)	97.2%	97.0%	97.1%	97.0%	GradCAM+++ LIME for Complete XAI

The proposed framework is naturally transferable to other crops and perishable commodities, as their fundamental design—consisting of CNN-based deep feature extraction, transformer-based attention modelling, multiclass SVM classification, and double-layer explainable AI integration—is not crop-dependent but is instead learnt and interpreted from high-resolution images of morphological and textural quality indicators. Through retraining of the pipeline with properly curated data, corresponding quality evaluation and recommendation systems may be constructed for a broad variety of perishable commodities like leafy crops (e.g., lettuce, kale), fruits (e.g., strawberries, tomatoes), and vegetables (e.g., cucumbers, bell peppers), where freshness loss occurs through similar visual indications like colour changes, surface injury, and texture modification. The rule-based recommendation system may be re-parameterised without much difficulty with confidence thresholds appropriate to each commodity’s perishability profile and each safety standard, respectively. This flexibility places SpinachXAI-Rec as a generalisable platform capable of accommodating smart, explainable, and scalable quality inspection across a variety of agri-food supply chains.

### Comparison of the state-of-the art models

SpinachNet-XAI framework’s only contribution and benefit over existing state-of-the-art methods for spinach and vegetable ripeness are found through the comparative study given in Table 15. Although a few models, e.g., Sankar Sennan et al. (2022) and Tapia Mendez et al. (2023), have achieved the same accuracy (high) as the current work (i) (all have ≥ 97%) (ii) predominantly employ the use of traditional CNNs or mere combinations (iii) have none of visual (and) explainable AI (XAI) (even) (iii) Pri, with the exception of Yıldırım & Yalıçın (2024) and Yıldırır & Yalıçın (2024), were not applied with the use of interpretability techniques, though the former had a hybrid-learning strategy used with their method, and the latter used a method which was a variation of the former (ResNet 101) but specific to spinach (not a ripeness method) and still does not offer transparency as a prediction. In contrast, He et al. (2024) put forward a non-destructive hyperspectral method which had biochemical understanding. But the intricacy, non-scalability, and absence of image-based CNN/XAI deflected away from this method. Unlike such methods, the herein-proposed SpinachNet-XAI has a distinction, as it encompasses a suggestion system that enables the individual to know the level of confidence the classification holds regarding the edibility of the spinach, a powerful combination of technologies—DenseNet121 + ViT-B/16 + SVM—and result-explanation tools (GradCAM +  + and LIME). This complete system, end-to-end, attains 97.2% classification accuracy and attends to vital gaps such as local–global interpretability, logic of consumption based on confidence, and real-world agri-clinical setting usability, which were absent in previous works. As such, the herein-proposed SpinachNet-XAI appears as a diagnostic system which is comprehensive, transparent, and smart, and which is specifically intended for the determination of spinach ripeness.

**Table 15:** Comparison of the state-of-the art models.

Author(s)	Year	Methodology	Accuracy	Explanation
Sankar Sennan et al	2022	Custom CNN on four leaf types	97.50%	High accuracy; limited dataset; lacks explainability (XAI)
Yıldırım & Yalçın	2024	ResNet101-based CNN for spinach freshness	89.40%	Moderate accuracy; lacks interpretability and hybrid ensemble
He et al	2024	Hyperspectral + DL classifiers (Spinach & Cabbage)	> 80%	Non-destructive analysis; requires expensive equipment; lacks CNN and explainability
Deep CNN + BiLSTM fusion	2024	CNN–BiLSTM hybrid for vegetable freshness	97.76%	Captures spatial–temporal features; computationally expensive; not spinach-specific; no visual XAI
Tapia Mendez et al	2023	MobileNetV2 ensemble for fruit & vegetable ripeness	97.86%	High accuracy; domain too broad; lacks spinach-specific dataset and explainability
Yuan & Chen	2024	GoogLeNet, DenseNet201, ResNeXt101 + PCA + SVM (feature fusion)	96.98%	Feature-based detection; efficient; no deep CNN retraining or XAI visual explanation
Koyama et al	2021	Color & local feature + SVM/ANN on smartphone images	84.00%	Non-deep learning; low accuracy; no CNN or XAI
Elumalai & Meganathan	2024	Orange pre-trained DL models + ML classifiers on spinach leaves	~ 99%	High accuracy; lacks interpretability and confidence-based classification
SpinachNet-XAI (Proposed)	2025	DenseNet121 + ViT-B/16 + SVM + GradCAM++ + LIME + Rule-based Logic	97.20%	High accuracy; complete explainability with XAI; confidence-based eatability recommendation provided

### Limitations and future work

We acknowledge important constraints regarding dataset representativeness, real-world resilience, and environmental variability. Even though our dataset is evenly balanced across six spinach classes, it is limited in geography and seasons; therefore, varietal variability, farm methods, post-harvest treatments, and retailer lighting/device variability are not comprehensively represented. We compensate in part through extensive augmentation (illumination, noise, blur), cross-model verification, and conservative recommender thresholds with dual-layer XAI checks, but remaining domain shift is conceivable under extreme illumination, motion blur, water droplets, or mixed-background clutter. We further investigated robustness through stress tests (e.g., down-sampling, contrast disturbances) and error studies, but broader external verification across sites, seasons, camera models (DSLR/mobile), and supply-chain nodes is needed. Future research will extend multi-site data capture, incorporate low-resource capture scenarios, introduce explicit calibration and uncertainty estimates, investigate domain adaptation (e.g., test-time adaptation, style transfer) and self-supervised pretraining for shift robustness, and implement post-deployment monitoring with human-in-the-loop escalation for borderline examples. Such efforts will enhance generalisability whilst retaining safety and trust in operational screening.

## Conclusion and future work

This study introduced SpinachNet-XAI, a comprehensive and comprehensible deep-learning system for the classification of the freshness of spinach into six categories—including fresh and non-fresh states of Malabar, red, and water spinach. Capitalising on a multi-stage framework, the system integrates data augmentation, classification by CNN (best individual result with DenseNet121), joint feature learning by Vision Transformers (ViT-B/16) as the feature extractor, and terminal ensemble multiclass SVM-based classification. Moreover, explainability is inherent through the incorporation of GradCAM++ and LIME, which offer both global visual as well as local feature-level explanations. A decisive rule-based recommender system transforms the values of confidence into comprehensible, health-orientated categories—Eatable, Eatable with Caution, or Not Eatable—thus enabling informed decisions by consumers, distributors, and agriculture experts. The performance systematically shows that the SpinachNet-XAI performs better than current literature through classification accuracy, interpretation, and real-world practicality. The system attains a robust F1-score of 0.97, an IoU of 0.89, and a Dice measure of 0.93, with the corresponding interpretable maps facilitating real-time judgement for the case of smart farming and monitoring of food safety.

Future efforts will involve the expansion of the system to other green leaf crops and highly perishable produce and the use of multi-modal data sources such as hyperspectral imaging, volatile compound sensor arrays, and humidity data. Moreover, real-time deployment via mobile/web applications, integration with edge AI devices for use out in the field, and the incorporation of user feedback into a semi-supervised learning feedback loop will be investigated. Long-term aims include the construction of a comprehensive AI platform for farm-to-fork freshness monitoring and food safety inspection, facilitating the achievement of wider aims in agri-clinical health monitoring and sustainable agriculture.

## Data Availability

All data used to support the findings of this study are included within the article.
